# Does chronic ankle instability patients lead to changes in biomechanical parameters associated with anterior cruciate ligament injury during landing? A systematic review and meta-analysis

**DOI:** 10.3389/fphys.2024.1428879

**Published:** 2024-08-29

**Authors:** Zhanyang He, Houwei Zhu, Binyong Ye, Zhe Zheng, Gongju Liu, Huiju Pan, Ronghua Liu

**Affiliations:** ^1^ College of Physical Education and Health Sciences, Zhejiang Normal University, Jinhua, China; ^2^ Scientific Research Center and Laboratory of Aquatic Sports Science of General Administration of Sports China, Zhejiang College of Sports, Hangzhou, China; ^3^ Shanghai University of Finance and Economics Zhejiang College, Jinhua, China

**Keywords:** chronic ankle instability, proximal, joint biomechanics, landing, anterior cruciate ligament

## Abstract

**Objective:**

This study aimed to determine if patients with chronic ankle instability (CAI) exhibit biomechanical changes associated with the increased risk of anterior cruciate ligament (ACL) injury during landing tasks.

**Study Design:**

This study was conducted through systematic review and meta-analysis.

**Data Sources:**

Searches were conducted in May 2024 across five electronic databases, including Web of Science, Scopus, PubMed, SPORTDiscus, and Cochrane Library.

**Eligibility Criteria:**

Studies were included if they (1) involved subjects with CAI and healthy controls and (2) assessed biomechanical variables such as ground reaction forces, joint angles, and joint torques.

**Results:**

Of the 675 identified studies, 171 were included in the review, and 13 were eligible for meta-analysis. The reviewed studies clearly defined research objectives, study populations, consistent participant recruitment, and exposures, and they used valid and reliable measures for outcomes. However, areas such as sample size calculation, study sample justification, blinding in assessments, and addressing confounders were not robust. This meta-analysis involved 542 participants (healthy group: n = 251; CAI group: n = 291). Compared with healthy individuals, patients with CAI exhibited a greater peak vertical ground reaction force (peak VGRF; SMD = 0.30, 95% CI: 0.07–0.53, *p* = 0.009), reduced hip flexion angles (SMD = −0.30, 95% CI: −0.51 to −0.17, *p* < 0.0001), increased trunk lateral flexion (SMD = 0.47, 95% CI: 0.05 to 0.9, *p* = 0.03), greater hip extension moments (SMD = 0.47, 95% CI: 0.09–0.84, *p* = 0.02), and increased knee extension moments (SMD = 0.39, 95% CI: 0.02–0.77, *p* = 0.04).

**Conclusion:**

During landing tasks, patients with CAI demonstrate increased hip extension moments and knee extension moments, decreased hip flexion angles, increased peak VGRF, and increased trunk lateral flexion angles. These biomechanical variables are associated with an elevated risk of ACL injuries.

**Systematic Review Registration:** Identifier CRD42024529349.

## 1 Introduction

Anterior cruciate ligament (ACL) injuries are among the most severe injuries in sports, which remarkably affect athletic performance (Davey et al., 2019). The incidence and cost of recovery from ACL injuries are high, with approximately 100,000 to 2,50,000 cases occurring annually in the United States alone (Hewett et al., 1999; Mancino et al., 2023). The estimated cost per injury is about $17,000, with an annual cost of approximately $646 million for surgeries and rehabilitation among females with ACL injuries (Hewett et al., 1999). Moreover, pain, functional limitations, and radiographic signs of osteoarthritis in the knee are evident 12–20 years post-injury (Lohmander et al., 2004; Myklebust and Bahr, 2005). The current paradigms for ACL injury mechanisms during landing include four main theories: Ligament dominance, Quadriceps dominance, Trunk dominance, and Leg dominance (Hewett et al., 2010). Based on these theories, previous scholars have identified eight biomechanical variables associated with ACL injury risk during landing (dynamics and kinematics in the horizontal, frontal, and sagittal planes, impact loads, and trunk movements) (Chou et al., 2023; Hewett et al., 2010). For instance, small knee and hip flexion angles in sagittal plane kinematics have been previously reported to correlate with a great risk of ACL injuries (Devita and Skelly, 1992; Lin et al., 2012; Padua et al., 2009; Trigsted et al., 2017) (Table 1). Identifying the biomechanical changes and influencing factors of individual ACL injury patterns is crucial for assessing functional capabilities and predicting subsequent injury risks post-return to sports.

**TABLE 1:** Grouping of biomechanical variables.

Sagittal Plane Kinematics	Hip flexion angle↓(Devita and Skelly, 1992; Trigsted et al., 2017)	Knee flexion angle↓(Devita and Skelly, 1992; Trigsted et al., 2017)	Ankle dorsiflexion angle↓(Boden et al., 2009; Padua et al., 2009)
Sagittal Plane Kinetics	Hip extension moment↑(Devita and Skelly, 1992; Trigsted et al., 2017)	Knee extension moment↑(Devita and Skelly, 1992; Trigsted et al., 2017)	
Frontal plane kinematics	Hip abduction angle↓(Hewett et al., 2005)	Knee abduction angle↑(Hewett et al., 2005)	
Frontal plane Kinetics	knee adduction moment↑(Lin et al., 2012)		
Horizontal plane kinematics	Hip Internal rotation angle↑(Devita and Skelly, 1992; Trigsted et al., 2017)	Knee Internal rotation angle↑(Devita and Skelly, 1992; Trigsted et al., 2017)	
Horizontal plane Kinetics	Hip Internal rotation Moment↑(Devita and Skelly, 1992; Trigsted et al., 2017)	Knee Internal rotation Moment↑(Devita and Skelly, 1992; Trigsted et al., 2017)	
Impact loading	Peak VGRF↑(Devita and Skelly, 1992)	Loading rate↑(Paterno et al., 2007)	
Trunk mechanism	Trunk flexion↓(Paterno et al., 2007)	Trunk lateral flexion↑(Kristianslund et al., 2012)	

Existing research suggests that chronic ankle instability (CAI) may be important predisposing factor for ACL injuries. Epidemiological surveys indicate that 52%–60% of patients with ACL injuries have a history of ankle sprains (Kramer et al., 2007; Söderman et al., 2001), and most ankle sprains develop into CAI (Theisen and Day, 2019). Studies by Theisen and Day also suggest that the altered lower limb biomechanics associated with CAI may increase the susceptibility to non-contact ACL injuries (Theisen and Day, 2019). The etiology of CAI is often attributed to injuries of the lateral ankle ligaments, primarily the anterior talofibular ligament, resulting from sprains, with or without damage to the calcaneofibular ligament (van Putte-Katier et al., 2015). In addition, CAI is a common sequela of recurrent ankle sprains (Gribble et al., 2016). About 73% of individuals with ankle sprains develop CAI (Theisen and Day, 2019). Patients with CAI often experience persistent symptoms of pain or weakness in the ankle (Gribble et al., 2013), which affects their quality of life and participation in physical activities (Arnold et al., 2011; Hubbard-Turner and Turner, 2015), as well as increases the risk of recurrent sprains and post-traumatic ankle osteoarthritis (Gribble et al., 2016; Moisan et al., 2017).

In recent years, CAI has received attention as a risk factor for ACL injuries during landing motions (Kikumoto et al., 2024). Current literature proposes that the mechanism of ACL injuries in patients with CAI is due to distal responses in the kinematic chain triggered by the ankle during landing tasks, which result in alterations in the proximal movement chain (knee joint, hip joint, and trunk) (Hertel and Corbett, 2019; Sueki et al., 2013). Munn et al. have shown that several somatosensory domains are impaired in patients with CAI, possibly due to ligament and joint proprioceptor damage during injury and potential neural damage post-ligament injury (Munn et al., 2010). Impairments in proprioceptors can lead to altered feedforward motor control mediated by spinal or supraspinal mechanisms, affecting centrally mediated motor control strategies (Brown et al., 2004; Caulfield and Garrett, 2002; Hass et al., 2010). These outcomes may lead to changes in the proximal movement chain to compensate for ankle instability and functional impairment (Bullock-Saxton, 1994; Koshino et al., 2013). Such compensatory phenomena in proximal joints could cause ACL injuries (Jeon et al., 2021; Xu et al., 2022). For example, Terada et al. found reduced knee flexion at the peak of the anterior tibial shear force in patients with CAI (Terada et al., 2014), and Jeon et al.’s meta-analysis identified greater vertical ground reaction forces (VGRF) upon landing in patients with CAI (Jeon et al., 2021), both results were associated with ACL injury risk. However, the aforementioned studies identified only a single biomechanical variable difference associated with ACL injuries, probably due to the limited outcome indicators set in previous research, which failed to report more biomechanical differences related to ACL injuries. Consequently, many studies based on similar measurements have produced inconsistent results, and the adoption of a single variable alone is insufficient to objectively describe the ACL injury mechanisms in CAI patients. The lack of combined analyses of variables related to ACL injuries, which limits the effective examination of how proximal adaptations caused by CAI lead to ACL injuries.

A 13-year epidemiological study revealed that landing maneuvers are a common cause of ACL injuries in sports with frequent jumping activities, such as basketball and volleyball (Agel et al., 2005). Krosshaug et al. (2006) analyzed videos of 39 ACL injuries during basketball games and found that most injuries occurred during non-contact landing maneuvers. The landing action, given its high impact and frequent occurrence, warrants particular attention (Kim et al., 2017; Konradsen and Voigt, 2002). Moreover, landing provides a controlled and easily standardizable experimental condition, facilitating accurate assessment and analysis of biomechanical parameters. Considering the commonality, representativeness, high impact, and robustness of landing measurements in ACL injury studies, this research focuses on the landing maneuvers of patients with CAI.

Therefore, a systematic review and meta-analysis were conducted to investigate whether biomechanical changes related to ACL injury risk are present in patients with CAI during landing tasks. Previous studies have found that patients with CAI often fail to effectively utilize the lower limb buffering mechanisms during landing owing to ankle instability (Jeon et al., 2021). Additionally, changes have been observed in the proximal movement chain of the lower limbs in CAI patients (Bullock-Saxton, 1994; Koshino et al., 2013). We hypothesized that compared with healthy individuals, those with CAI will exhibit a greater peak VGRF and an upright body posture (i.e., reduced knee and hip flexion), leading to an increased risk of ACL injury.

## 2 Methods

This review was conducted in accordance with the PRISMA guidelines for systematic reviews and meta-analyses. It was prospectively registered in the international database for systematic reviews. (PROSPERO registration number: CRD42024529349).

### 2.1 Identification of ACL injury-related variables

Four theories based on the mechanism of landing ACL injury (Ligament dominance; Quadriceps dominance; Trunk dominance; leg dominance) (Hewett et al., 2010), and previous prospective and case-control studies identified eight biomechanical variables associated with ACL injury risk (Chou et al., 2023; Hewett et al., 2010). These variables have been adopted in several high-quality studies (Chou et al., 2023), thereby proving their reliability. Table 1 illustrates the detrimental directionality of biomechanical variables in each construct (high injury risk). Following data extraction, clarifying which outcome indicators are used for correlation analysis is crucial, serving as the bridge to determine whether CAI is associated with ACL injuries.

### 2.2 Data sources and search strategy

In May 2024, a systematic search was conducted across five electronic databases: Web of Science, Scopus, PubMed, SPORTDiscus, and Cochrane Library. Keywords related to CAI and biomechanical variables were used to identify relevant articles. Keywords for each category were combined using the Boolean operator “OR” and then combined across categories using “AND” for each database search. There was no restriction on publication date. Boolean logic was utilized for all database searches: (chronic ankle instability OR ankle instability OR functional ankle instability OR mechanical ankle instability OR CAI OR FAI OR MAI) AND (lower limb OR lower extremity OR hip OR knee OR ankle OR trunk) AND (kinematic OR kinetics OR biomechanics OR Moment OR Torque OR dynamic) AND [1) stop jump: (stop jump OR stop-jump OR stop jumping OR stop-jumping), 2) landing: (land OR landing OR jump land OR jump landing OR jump-land OR jump-landing OR drop-vertical jump OR single-leg landing OR single-leg land OR jump OR jumping)]. The detailed search strategy is presented in Table 2.

**TABLE 2:** Search strategy for each database.

Database	Web of Science	Scopus	PubMed	SPORTDiscus	Cochrane Library
Applied database fields used During The search	Topic (Title, abstract, author, keywords, and Keywords Plus)	Title Abstract, keyword	Title, Abstract	Title, Abstract	Title Abstract, keyword
Restrictions for the search	None				
Examples of the strategy Web of Science	(((TS=(chronic ankle instability OR ankle instability OR functional ankle instability OR mechanical ankle instability OR CAI OR FAI OR MAI)) AND TS=(lower limb OR lower extremity OR hip OR knee OR ankle OR trunk)) AND TS=(kinematic OR kinetics OR biomechanics OR Moment OR Torque OR dynamic OR angles OR moments OR forces OR ground reaction force OR GRF OR displacement)) AND TS=(stop jump OR stop-jump OR stop jumping OR stop-jumping OR land OR landing OR jump land OR jump landing OR jump-land OR jump-landing OR drop-vertical jump OR single-leg landing OR single-leg land OR jump OR jumping)				
Examples of the strategy Scopus	(TITLE-ABS-KEY ( “chronic ankle instability” OR “ankle instability” OR “functional ankle instability” OR “mechanical ankle instability” OR “CAI” OR “FAI” OR “MAI”) AND TITLE-ABS-KEY (“lower limb” OR “lower extremity” OR “hip” OR “knee” OR “ankle” OR “trunk”) AND TITLE-ABS-KEY (“kinematic” OR “kinetics” OR “biomechanics” OR “Moment” OR “Torque” OR “dynamic” OR “angles” OR “moments” OR “forces” OR “ground reaction force” OR “GRF” OR “displacement”) AND TITLE-ABS-KEY (“stop jump” OR “stop-jump” OR “stop jumping” OR “stop-jumping” OR “land” OR “landing” OR “jump land” OR “jump landing” OR “jump-land” OR “jump-landing” OR “drop-vertical jump” OR “single-leg landing” OR “single-leg land” OR “jump” OR “jumping”) )				
Examples of the strategy PubMed	(((chronic ankle instability [Title/Abstract] OR ankle instability [Title/Abstract] OR functional ankle instability [Title/Abstract] OR mechanical ankle instability [Title/Abstract] OR CAI [Title/Abstract] OR FAI [Title/Abstract] OR MAI [Title/Abstract]) AND (lower limb [Title/Abstract] OR lower extremity [Title/Abstract] OR hip [Title/Abstract] OR knee [Title/Abstract] OR ankle [Title/Abstract] OR trunk [Title/Abstract])) AND (kinematic [Title/Abstract] OR kinetics [Title/Abstract] OR biomechanics [Title/Abstract] OR Moment [Title/Abstract] OR Torque [Title/Abstract] OR dynamic [Title/Abstract] OR angles [Title/Abstract] OR moments [Title/Abstract] OR forces [Title/Abstract] OR ground reaction force [Title/Abstract] OR GRF [Title/Abstract] OR displacement [Title/Abstract])) AND (stop jump [Title/Abstract] OR stop-jump [Title/Abstract] OR stop jumping [Title/Abstract] OR stop-jumping [Title/Abstract] OR land [Title/Abstract] OR landing [Title/Abstract] OR jump land [Title/Abstract] OR jump landing [Title/Abstract] OR jump-land [Title/Abstract] OR jump-landing [Title/Abstract] OR drop-vertical jump [Title/Abstract] OR single-leg landing [Title/Abstract] OR single-leg land [Title/Abstract] OR jump [Title/Abstract] OR jumping [Title/Abstract])				
Examples of the strategy SPORTDiscus	TI (chronic ankle instability OR ankle instability OR functional ankle instability OR mechanical ankle instability OR CAI OR FAI OR MAI) AND TI (lower limb OR lower extremity OR hip OR knee OR ankle OR trunk) AND TI (kinematic OR kinetics OR biomechanics OR Moment OR Torque OR dynamic OR angles OR moments OR forces OR ground reaction force OR GRF OR displacement) AND TI (stop jump OR stop-jump OR stop jumping OR stop-jumping OR land OR landing OR jump land OR jump landing OR jump-land OR jump-landing OR drop-vertical jump OR single-leg landing OR single-leg land OR jump OR jumping)				
	AB (chronic ankle instability OR ankle instability OR functional ankle instability OR mechanical ankle instability OR CAI OR FAI OR MAI) AND AB (lower limb OR lower extremity OR hip OR knee OR ankle OR trunk) AND AB (kinematic OR kinetics OR biomechanics OR Moment OR Torque OR dynamic) AND AB (stop jump OR stop-jump OR stop jumping OR stop-jumping OR land OR landing OR jump land OR jump landing OR jump-land OR jump-landing OR drop-vertical jump OR single-leg landing OR single-leg land OR jump OR jumping)				
Examples of the strategy Cochrane Library	chronic ankle instability OR ankle instability OR functional ankle instability OR mechanical ankle instability OR CAI OR FAI OR MAI in Title Abstract Keyword AND lower limb OR lower extremity OR hip OR knee OR ankle OR trunk in Title Abstract Keyword AND kinematic OR kinetics OR biomechanics OR Moment OR Torque OR dynamic OR angles OR moments OR forces OR ground reaction force OR GRF OR displacement in Title Abstract Keyword AND stop jump OR stop-jump OR stop jumping OR stop-jumping OR land OR landing OR jump land OR jump landing OR jump-land OR jump-landing OR drop-vertical jump OR single-leg landing OR single-leg land OR jump OR jumping in Title Abstract Keyword - (Word variations have been searched)				

### 2.3 Inclusion criteria

The search results were independently screened by two reviewers (Z.H., B.Y.) on the basis of the predetermined inclusion and exclusion criteria, with disputes resolved by the corresponding author (H.Z.) if necessary. Studies were included if they met the following criteria: In accordance with the PICOS guidelines, studies meeting the following criteria were included: P: The study population consists of individuals with CAI and healthy control subjects. I: Participants perform landing tasks as an intervention measure. C: CAI patients are compared with a healthy control group. O: The reported outcomes are biomechanical variables, such as ground reaction forces, joint angles, and joint torques. S: Cross-sectional studies are included. Exclusion criteria included the following: 1) variables unrelated to ACL injury outcome indicators (Table 1); 2) task location is not on a flat surface (differences in biomechanics of landing on flat and sloped surfaces may affect the robustness of study results. Additionally, sloped landings are uncommon in real-life activities.); 3) studies employing one-dimensional statistical parametric mapping analysis (Data cannot be pooled for meta-analysis); 4) review articles, editorials, speeches, commentaries, abstracts, case studies, surgical procedures, and non-peer-reviewed articles.

### 2.4 Study selection

Titles and abstracts were screened by two independent reviewers, with any discrepancies resolved through consultation with a third author. If eligibility could not be determined from the title and abstract, then full texts were obtained and reviewed. Cohen’s kappa coefficient (κ) and percentage agreement were used to assess inter-reviewer consistency. κ was interpreted on the basis of Landis and Koch’s standards: Values less than 0 indicated no agreement, 0–0.20 indicated slight agreement; 0.21–0.40 indicated fair agreement; 0.41–0.60 indicated moderate agreement; 0.61–0.80 indicated substantial agreement; and 0.81–1 indicated almost perfect agreement (Landis and Koch, 1977). Full texts of all eligible studies were retrieved, and data such as demographics (e.g., gender and age), sample size, and biomechanical variable results (e.g., standardized ground reaction forces, joint angles, and standardized joint torques) were extracted.

### 2.5 Quality assessment and analysis

The quality of included studies was assessed using the NIH quality assessment tool for observational cohort and cross-sectional studies. Two researchers (Z.H. and B.Y.) independently evaluated the quality of each study, with any disagreements resolved through discussion until consensus was reached.

### 2.6 Publication bias

Publication bias was assessed using funnel plots and Egger’s regression test. Visual inspection of funnel plot symmetry indicated an unbiased range of effect sizes among the included studies. Asymmetry in the funnel plot suggested bias toward specific effect sizes or sample sizes. Egger’s regression test quantified bias using the effect size of each included study, with a *p*-value less than 0.05 indicating significant publication bias (Borenstein et al., 2021).

### 2.7 Sensitivity analysis

Sensitivity analyses were conducted on studies included in the meta-analysis to determine if the exclusion of influential studies affected the overall effect results. Thus, the most influential studies were excluded from the meta-analysis and analysis was repeated.

### 2.8 Data extraction and statistical analysis

Data were extracted by two authors (Z.H., B.Y.) using standardized forms from each included study. The extracted data were as follows: 1) sample description, encompassing the sample size, age, and gender; 2) information related to CAI, including the definition of CAI and scores from the Cumberland Ankle Instability Tool; 3) biomechanical variables related to outcome indicators (Table 1); 4) standardized results, including means, SDs, *p*-values, and standardized difference in effect sizes; 5) task conditions, specifically limited to landing tasks performed on flat surfaces. The data extracted from all included studies were then synthesized for comprehensive analysis. Statistical analysis compared biomechanical variables between patients with CAI and healthy individuals as the effect sizes for meta-analysis. To avoid errors caused by multiple biomechanical variables within each structure influencing the final outcome, we conducted separate meta-analyses for biomechanical outcome indicators within each structure (e.g., separate meta-analyses for Trunk flexion and trunk lateral flexion within the trunk mechanism structure). Additionally, to prevent reliance on effect sizes from the same study, we averaged the effect sizes based on specific task conditions (e.g., averaging the parameters of a biomechanical variable at different stages during a landing task) (Borenstein et al., 2021).

The overall effect size for each variable was calculated using sample size, means, standard deviations, mean differences, and *p*-values. Meta-analyses were performed using Review Manager V.5.3 (Copenhagen, Denmark: The Nordic Cochrane Centre, The Cochrane Collaboration, 2014). Owing to variations in drop platform heights and the differences between single and double foot landings among the included studies, ensuring complete consistency across multicenter and multiprotocol studies is challenging. Therefore, this study calculated the effect size of each biomechanical variable using the standardized mean difference (SMD) with a 95% confidence interval (CI). A random effect model was employed. Heterogeneity among the included studies was assessed using the I^2^ statistic.

## 3 Results

### 3.1 Study selection and characteristics

**FIGURE 1:**
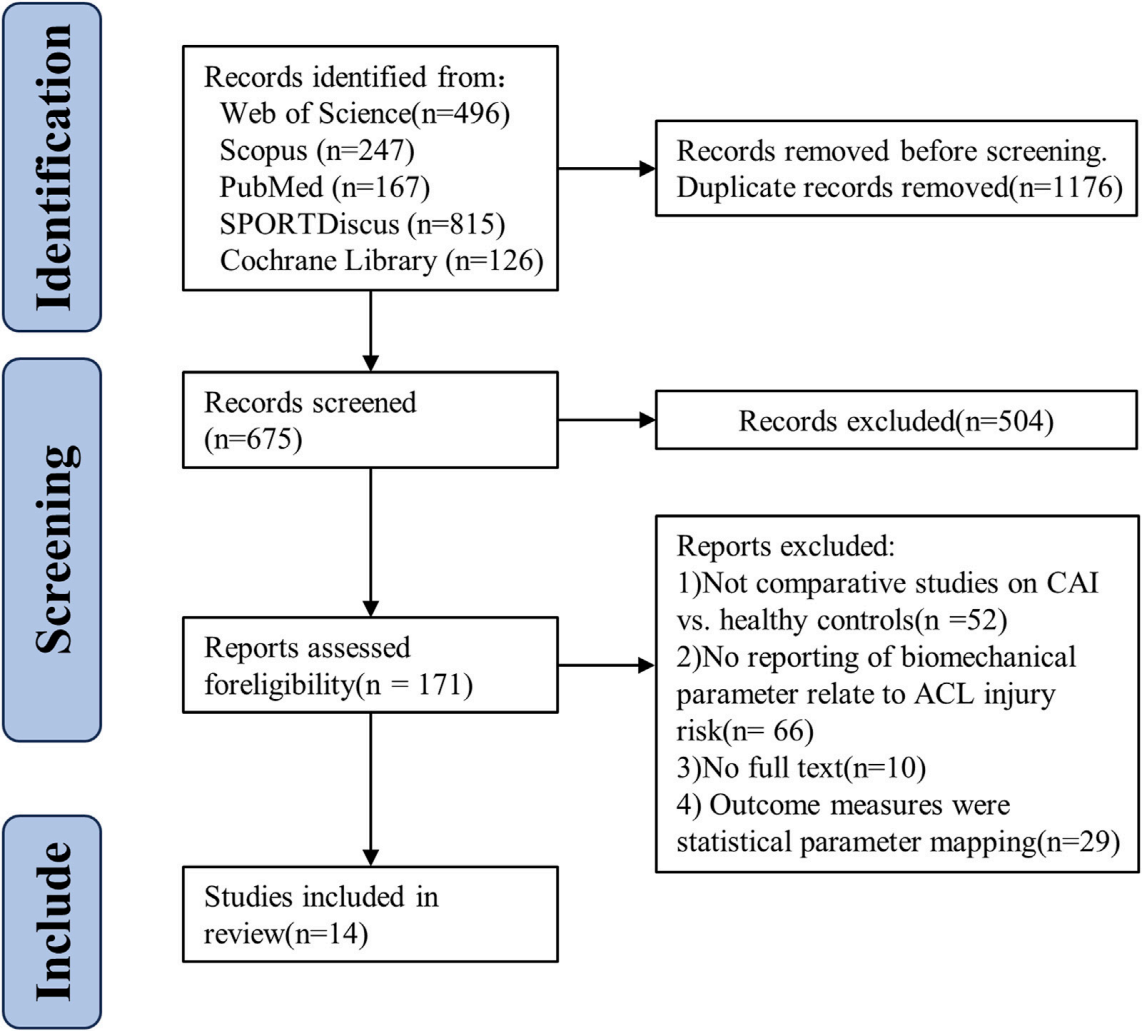
Flow chart of systematic reviews and meta-analyses.

An initial search across five databases yielded 1,615 articles. After duplicates were removed, 675 studies remained. Title and abstract screening by two reviewers eliminated 504 studies (consistency rate = 97.62%, κ = 0.49), and 171 studies were assessed for eligibility through full-text review. After this review, 157 studies were excluded, leaving 14 studies included in the meta-analysis (consistency rate = 100%, κ = 1). The detailed exclusion process at each stage is depicted in Figure 1. A total of 564 participants were involved in this study (healthy group: n = 262; CAI group: n = 302), with detailed characteristics of the included studies provided in Table 3. For variables related to ACL injury, seven studies were included on peak VGRF (Caulfield and Garrett, 2004; De Ridder et al., 2015; Jeon and Park, 2021; Lee et al., 2017; Watabe et al., 2022; Watanabe et al., 2021; Zhang et al., 2012), three studies on loading rate (De Ridder et al., 2015; Jeon and Park, 2021; Lee et al., 2017); nine and ten studies on hip flexion angle (Brown et al., 2011; Caulfield and Garrett, 2004; Gribble and Robinson, 2009; Han et al., 2023; Jeon and Park, 2021; Sagawa et al., 2024; Terada et al., 2015; Terada et al., 2014; Watabe et al., 2022; Watanabe et al., 2021) and knee flexion angle (Brown et al., 2011; Caulfield and Garrett, 2004; Gribble and Robinson, 2009; Han et al., 2023; Jeon and Park, 2021; Sagawa et al., 2024; Terada et al., 2015; Terada et al., 2014; Watabe et al., 2022; Watanabe et al., 2021), respectively. Ankle flexion angle is covered in 10 studies (Gribble and Robinson, 2009; Han et al., 2023; Jeon and Park, 2021; Kipp and Palmieri-Smith, 2012; Lee et al., 2017; Sagawa et al., 2024; Terada et al., 2015; Watabe et al., 2022; Watanabe et al., 2021; Zhang et al., 2012); and two studies each on hip extension moment (Jeon and Park, 2021; Watanabe et al., 2021) and knee extension moment (Jeon and Park, 2021; Watanabe et al., 2021). Four studies on hip abduction angle (Brown et al., 2011; Jeon and Park, 2021; Sagawa et al., 2024; Terada et al., 2015) and three studies on knee abduction angle (De Ridder et al., 2015; Jeon and Park, 2021; Lee et al., 2017); two studies on trunk flexion (Brown et al., 2011; Watabe et al., 2022) and two studies on trunk lateral flexion (Brown et al., 2011; Watabe et al., 2022). No other biomechanical variables related to ACL injuries were reviewed in this study (Table 4).

**TABLE 3:** Outcome indicators determined.

Sagittal Plane Kinematics of the Lower Limb	Hip flexion angle↓(Devita and Skelly, 1992; Trigsted et al., 2017)	Knee flexion angle↓(Devita and Skelly, 1992; Trigsted et al., 2017)	Ankle dorsiflexion angle↓(Boden et al., 2009; Padua et al., 2009)
Sagittal Plane Kinetics of the Lower Limb	Hip extension moment↑(Devita and Skelly, 1992; Trigsted et al., 2017)	Knee extension moment↑(Devita and Skelly, 1992; Trigsted et al., 2017)	
Frontal plane kinematics of the Lower Limb	Hip abduction angle↓(Hewett et al., 2005)	Knee abduction angle↑(Hewett et al., 2005)	
Impact loading	Peak VGRF↑(Devita and Skelly, 1992)	Loading rate↑(Paterno et al., 2007)	
Trunk mechanism	Trunk flexion↓(Paterno et al., 2007)	Trunk lateral flexion↑(Kristianslund et al., 2012)	

**TABLE 4:** Study characteristics and participant demographics.

First Author (year)	Test method	Study type	Population (total/male/female)	Outcome measures
Watanabe et al. (2021)	single-leg landing (0.3 m,0.4 m,0.5 m)	cross-sectional	26 Competitive collegiate athletes; Control group = 13/7/4 Age = 20.6 ± 2.1 CAI group = 13/7/4 Age = 21.6 ± 1.6	*Kinematics* Peak knee Flexion angle (0.3 m,0.4 m,0.5 m) Peak hip flexion angle (0.3 m,0.4 m,0.5 m) *Kinetics* Peak knee extension moment (0.3 m,0.4 m,0.5 m) Peak hip extension moment (0.3 m,0.4 m,0.5 m) *Impact Loading* Peak VGRF (0.3 m,0.4 m,0.5 m)
Gribble and Robinson (2009)	double-leg take-off jump with a landing on a single limb	cross-sectional	Control group = 19/10/9 Age = 23.1 ± 3.9 CAI group = 19/10/9 Age = 20.3 ± 2.9	*Kinematics* Peak knee flexion angle (Injured side) Peak hip flexion (Injured side)
Zhang et al. (2012)	drop landing (0.6 m)	cross-sectional	Control group = 10Age = 24.1 (5.4) CAI group = 10 Age = 24.8 (5.7)	*Impact loading* 2nd peak vertical GRF
Terada et al. (2014)	vertical stop jump (50% of Vertmax)	case–control experiment design	Thirty-eight physically active participants Control group = 19/10/9 Age = 21.32 ± 4.04) CAI group = 19/10/9 Age = 20.11 ± 1.63	*Kinematics* Knee sagittal plane angle at Peak anterior tibial shear force (ATSF) Hip sagittal plane angle at Peak ATSF
Lee et al. (2017)	single-leg drop landing	Controlled laboratory	28 competitive taekwondo athletes Control group = 14/14 Age = 21.21 ± 2.08 CAI group = 14/14 Age = 20.07 ± 0.27	*Impact loading* Peak VGRF Loading rate
De Ridder et al. (2015)	Vertical drop (0.4 m)	cross-sectional	Control group = 30/12/18 Age = 25.7 ± 1.8 CAI group = 38/19/19 Age = 22.1 ± 3.4	*Impact Loading* Peak vertical GRF Loading rate
Caulfield and Garrett (2004)	single leg vertical drop (0.4 m)	cross-sectional	Control group = 10/10 Age = 22.6 ± 4.6 FAI group = 14/14 Age = 26.6 ± 6.3	*Impact loading* Peak vertical GRF
Jeon and Park (2021)	Drop landing (0.3 m)	cross-sectional	Control group = 18/18 Age = 20.22 ± 2.29 CAI group = 16/16 Age = 20.19 ± 1.47	*Kinematics* Hip flexion joint angle; hip abduction joint angle; Knee Flexion joint angle Knee Valgus joint angle *Kinetics* Hip flexion Joint moment Knee flexion Joint moment *Impact loading* Max vGRF Loading rate
Watabe et al. (2022)	proactive condition (single-leg landings) reactive condition (side-step cutting, 60° side-step cutting, single-leg landing, and forward stepping)	cross-sectional	28 physically active individuals; Control group = 14/Age = 21.5 ± 1.3 CAI group = 14/Age = 21.4 ± 1.4	*Kinematics* Maximum Right lateral trunk flexion in (PRO,REA) Maximum Trunk flexionin in (PRO,REA) Maximum Hip flexion in (PRO,REA) Maximum Knee flexion in (PRO,REA) Maximum Ankle dorsiflexion in (PRO,REA) *Impact loading* Vertical ground reaction force in (PRO,REA)
Brown et al. (2011)	single-leg landing in the anterior, lateral, and medial directions (50% of Vertmax)	cross-sectional	68 recreationally active participants Control group = 24/12/12male’s Age = 19.8 ± 13/female’s Age = 20.2 ± 1.0 MAI group = 21/8/13 male’s Age = 18.6 ± 3.3/female’s Age = 19.9 ± 1.0 FAI group = 23/11/12 male’s Age = 20.5 ± 1.7/female’s Age = 20.1 ± 1.5	*Kinematics* Knee flexion–extension angle Knee abduction–adduction angle Hip flexion angle Hip abduction–adduction angle Trunk flexion angle Trunk lateral flexion angle
Han et al. (2023)	single-leg drop-landing	cross-sectional	44 physically active individuals Control group = 22/11/11 Age = 23.4 ± 2.6) CAI group = 22/11/11 Age = 23.4 ± 2.4	*Kinematics* Knee flexion angle at initial contact in (Anticipated,Unanticipated) Maximum knee flexion angle in (Anticipated,Unanticipated) Knee displacement in (Anticipated,Unanticipated) Hip flexion angle at initial contact in (Anticipated,Unanticipated) Maximum hip flexion angle in (Anticipated,Unanticipated) Hip displacement in (Anticipated,Unanticipated)
Terada et al. (2015)	single-leg drop landing (0.3 m) conditions: (1) looking-down and (2) looking-up	Controlled laboratory	Thirty-eight physically active participants Control group = 19/6/13 Age = 20.58 ± 2.32 CAI group = 19/11/8 Age = 21.68 ± 4.82	*Kinematics* Hip sagittal plane angle at initial contact (looking-up,looking-down) Hip frontal plane at initial contact (looking-up,looking-down) Knee sagittal plane at initial contact (looking-up,looking-down) Knee frontal plane at initial contact (looking-up,looking-down)
Sagawa et al. (2024)	Single-leg Lateral Drop Landing (0.2 m)	cross-sectional	Control group = 19/19/0 Age = 23.9 ± 2.8 CAI group = 19/19/0 Age = 25.3 ± 2.9	*Kinematics* Hip Flexion maximum angle during 200 m interval post-landing Hip Abduction maximum angle during 200 m interval post-landing Knee Flexion maximum angle during 200 m interval post-landing

### 3.2 Quality assessment

The results of the quality assessment tool developed by NIH for observational cohorts and cross-sectional studies ranged from five to 9 (Table 5). The evaluation results highlight some apparent strengths and limitations of the included literature. The strengths lie in the studies having clear objectives, defined study populations, and consistent participant recruitment, and using valid and reliable methods for exposure definition and outcome measurement. These aspects are important indicators of high research quality, suggesting that the design and execution of the studies were successful in these respects. However, some shortcomings remain. Only two studies calculated the sample size and determined an appropriate study sample size, which could affect the statistical power of the results and the reliability of the conclusions. Furthermore, the lack of assessment of blinding implementation and insufficient justification for confounding variables could lead to bias and errors in the study outcomes.

**TABLE 5:** Methodological quality score using the NIH quality assessment tool of relevant studies.

First Author (year)	Q1	Q2	Q3	Q4	Q5	Q6	Q7	Q8	Q9	Q10	Q11	Q12	Q13	Q14	Total (Yes)
Watanabe et al. (2021)	Yes	Yes	CD	Yes	Yes	No	No	NA	Yes	No	Yes	No	NA	No	6
Kipp and Palmieri-Smith (2012)	Yes	Yes	CD	Yes	No	No	No	NA	Yes	No	Yes	No	NA	No	5
Gribble and Robinson (2009)	Yes	Yes	CD	Yes	No	No	No	NA	Yes	No	Yes	No	NA	No	5
Zhang et al. (2012)	Yes	Yes	CD	Yes	No	No	No	NA	Yes	No	Yes	No	NA	No	5
Terada et al. (2014)	Yes	Yes	CD	Yes	No	No	No	NA	Yes	No	Yes	No	NA	No	5
Lee et al. (2017)	Yes	Yes	CD	Yes	No	No	No	NA	Yes	No	Yes	No	NA	No	5
De Ridder et al. (2015)	Yes	Yes	CD	Yes	No	No	No	NA	Yes	No	Yes	No	NA	No	5
Caulfield and Garrett (2004)	Yes	Yes	CD	Yes	No	No	No	NA	Yes	No	Yes	No	NA	No	5
Jeon and Park (2021)	Yes	Yes	CD	Yes	No	No	No	NA	Yes	No	Yes	No	NA	No	5
Watabe et al. (2022)	Yes	Yes	CD	Yes	No	No	No	NA	Yes	No	Yes	No	NA	No	5
Han et al. (2023)	Yes	Yes	CD	Yes	No	No	No	NA	Yes	No	Yes	No	NA	No	5
Terada et al. (2015)	Yes	Yes	CD	Yes	No	No	No	NA	Yes	No	Yes	No	NA	No	5
Brown et al. (2011)	Yes	Yes	CD	Yes	No	No	No	NA	Yes	No	Yes	No	NA	No	5
Sagawa et al. (2024)	Yes	Yes	CD	Yes	Yes	No	No	NA	Yes	No	Yes	No	NA	No	6

Q1: was the research question or objective in this paper clearly stated?, Q2: was the study population clearly specified and defined?, Q3: was the participation rate of eligible persons at least 50%?, Q4: were all the subjects selected or recruited from the same or similar populations (including the same time period)? Were inclusion and exclusion criteria for being in the study prespecified and applied uniformly to all participants?, Q5: was a sample size justification, power description, or variance and effect estimates provided?, Q6: for the analyses in this paper, were the exposure(s) of interest measured prior to the outcome(s) being measured?, Q7: was the timeframe sufficient so that one could reasonably expect to see an association between exposure and outcome if it existed?, Q8: for exposures that can vary in amount or level, did the study examine different levels of the exposure as related to the outcome (e.g., categories of exposure, or exposure measured as continuous variable)?, Q9: were the exposure measures (independent variables) clearly defined, valid, reliable, and implemented consistently across all study participants?, Q10: was the exposure(s) assessed more than once over time?, Q11: were the outcome measures (dependent variables) clearly defined, valid, reliable, and implemented consistently across all study participants?, Q12: were the outcome assessors blinded to the exposure status of participants?, Q13: was loss to follow-up after baseline 20% or less?, Q14: were key potential confounding variables measured and adjusted statistically for their impact on the relationship between exposure(s) and outcome(s)? CD: cannot determine,NA: notapplicable.

### 3.3 Publication bias

Publication bias was detected in the trunk flexion angle (*p* = 0.007). No publication bias was found for the remaining variables (Supplementary Appendix S1).

### 3.4 Heterogeneity

**FIGURE 2:**
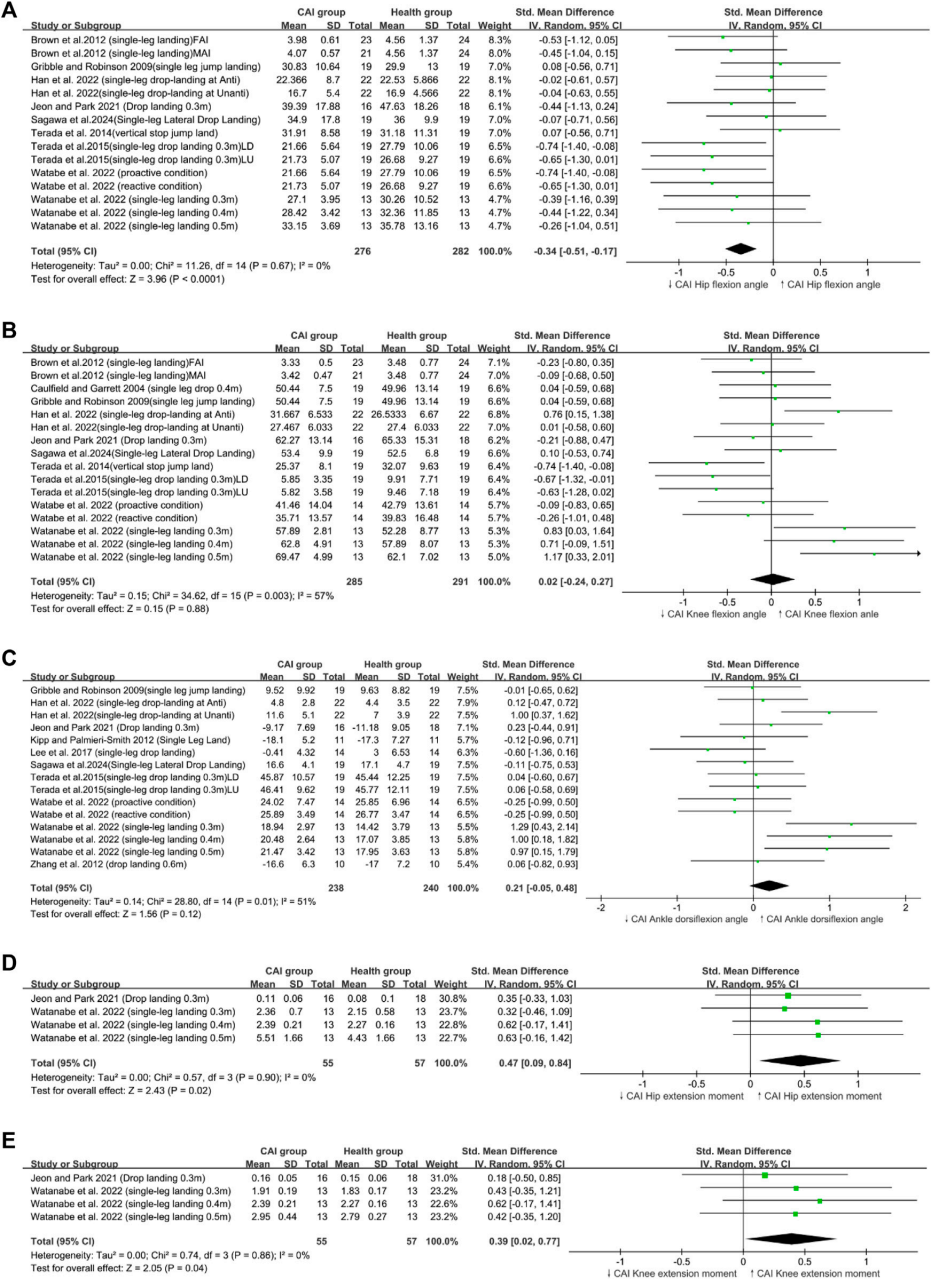
Sagittal Plane biomechanical parameters; **(A)** Hip flexion angle, **(B)** Knee flexion angle, **(C)** Ankle dorsiflexion angle **(D)** Hip extension moment, **(E)** Knee extension moment.

**FIGURE 3:**
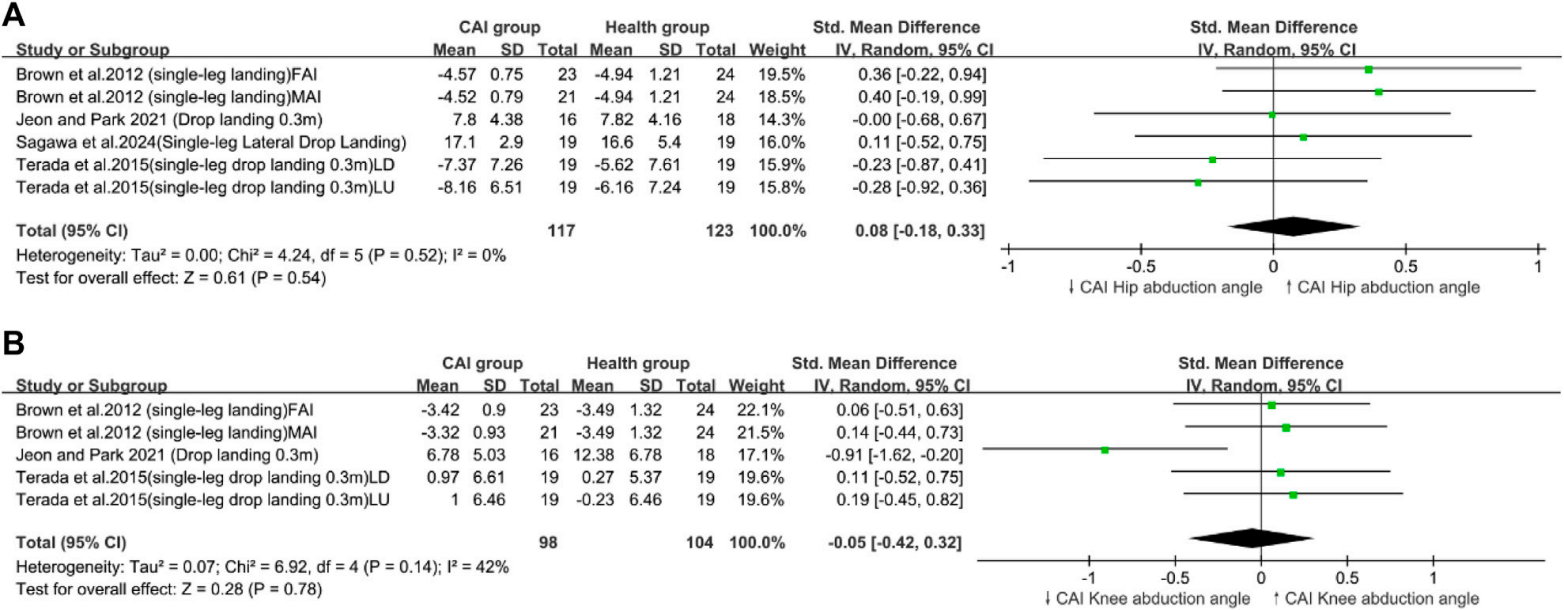
Frontal plane kinematics parameters; **(A)**: Hip abduction angle, **(B)**: Knee abduction angle

**FIGURE 4:**
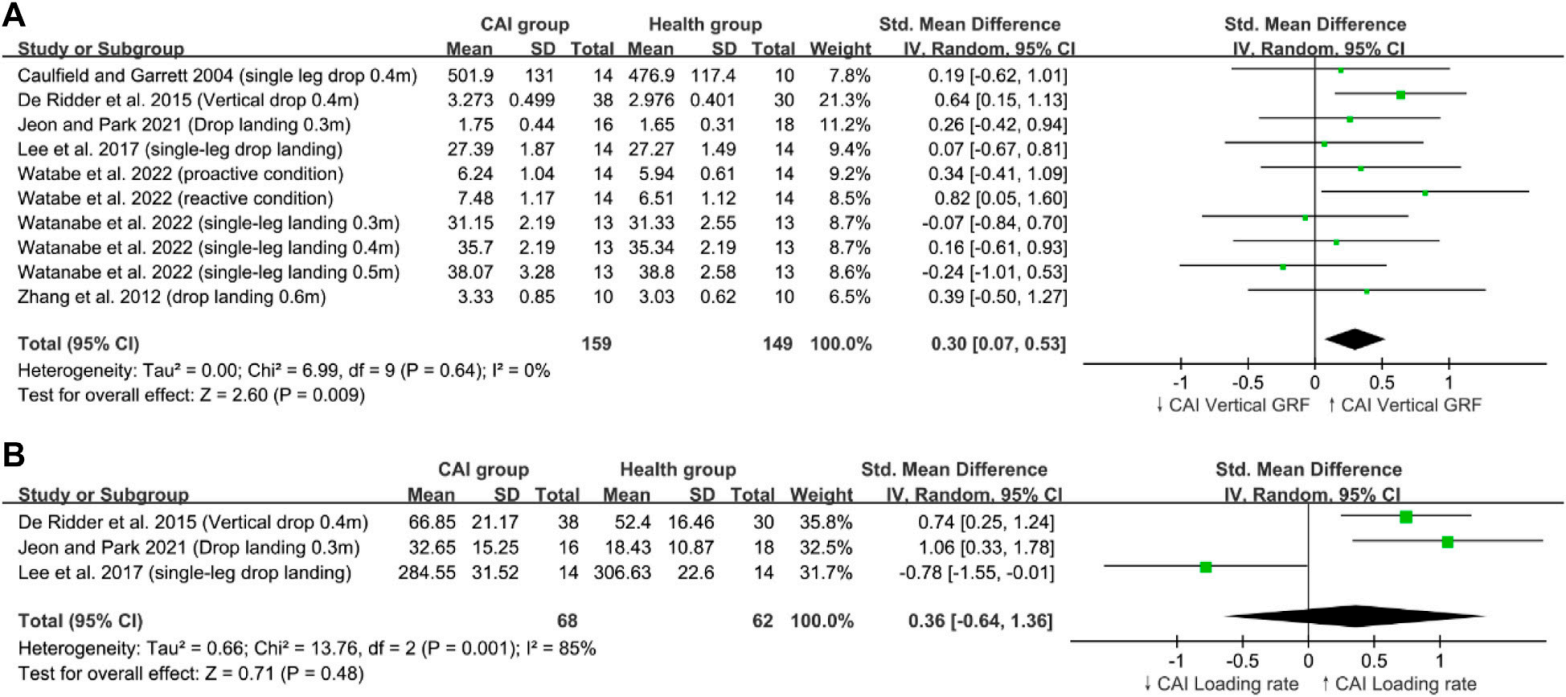
Impact loading; **(A)**: Peak VGRF, **(B)**: Loading rate.

Heterogeneity was present in the ankle dorsiflexion angle (Figure 2C), loading rate (Figure 3A), knee flexion angle (Figure 2B), and trunk flexion (Figure 4B).

### 3.5 Study findings

Our research findings indicated that patients with CAI exhibited a greater maximum (peak VGRF; SMD = 0.30, 95% CI: 0.07–0.53, *p* = 0.009), a reduced hip flexion angle (SMD = −0.34,95% CI: −0.53 to −0.19, *p* < 0.0001), (Figure 4A) and an increased trunk lateral flexion angle (SMD = 0.47,95% CI: 0.05–0.9, *p* = 0.03) during landing tasks. In Addition, these patients showed increased hip extension moments (SMD = 0.47,95% CI: 0.09–0.84, *p* = 0.02) and greater knee extension moments (SMD = 0.39,95% CI: 0.02–0.77, *p* = 0.04) (Figures 2C, D) compared with healthy controls. By contrast, no significant differences in the loading rate, knee flexion angle, trunk flexion angle, knee abduction angle (Figure 3B), ankle dorsiflexion angle (Figure 2E) and hip abduction angle were found between patients with CAI and healthy individuals. The sensitivity analysis results reveal that the outcomes for Knee extension moment and Loading rate are significantly influenced by individual studies (Supplementary Appendix S4). Specifically, removing the study by Jeon and Park (2021) led to a significant change in the *p*-value for Knee extension moment (*p* < 0.05), and removing Lee et al. (2017) similarly affected the *p*-value for Loading rate (*p* < 0.05). Due to the limited number of studies included, further subgroup analysis to explore these differences was not feasible. This limitation highlights the need for cautious interpretation of our conclusions. Additionally, the results for other variables remained consistent and unchanged in the sensitivity analysis.

## 4 Discussion

This systematic review aimed to determine whether changes in lower limb biomechanics during landing tasks in the CAI population increase the risk of ACL injury compared with that in a healthy population. This meta-analysis included data from 14 studies (Brown et al., 2011; Caulfield and Garrett, 2004; De Ridder et al., 2015; Gribble and Robinson, 2009; Han et al., 2023; Jeon and Park, 2021; Kipp and Palmieri-Smith, 2012; Lee et al., 2017; Sagawa et al., 2024; Terada et al., 2015; Terada et al., 2014; Watabe et al., 2022; Watanabe et al., 2021; Zhang et al., 2012), which compared the landing biomechanics between patients with CAI and healthy individuals. Our findings indicated that patients with CAI exhibited a greater peak VGRF during landing compared with healthy individuals, which was consistent with our initial hypothesis. A decreased hip flexion angle was also observed, partially supporting our hypothesis that the body was upright, although no significant difference was found in knee flexion angles. Additionally, an increased trunk lateral flexion, an increased hip extension moment, and an increased knee extension moment were observed in patients with CAI.

### 4.1 Sagittal plane kinematics and kinetics of the lower limb

Our study revealed that individuals with CAI landed with decreased hip flexion angles and increased knee and hip extension moments. However, contrary to our expectations, patients with CAI did not exhibit reduced knee flexion angles or dorsiflexion angles. This finding is also in contrast to the findings of Chan et al. (2022) and Aaron and James (Theisen and Day, 2019), who reported reduced knee flexion and ankle dorsiflexion in similar contexts (Chan et al., 2022). This inconsistency may stem from our meta-analysis incorporating anticipated and unanticipated landing conditions (Han et al., 2023; Watabe et al., 2022) and changes in visual focus during landing (Terada et al., 2015). Analyzing multiple states together led to high heterogeneity, but this result was considered acceptable because it reflected the different dynamic states encountered during athletic activities. Moreover, our findings suggested that patients with CAI developed neuromuscular feedforward mechanisms that resulted in upright postures and increased hip extension moments during landing, thereby supporting our study outcomes (Figure 2) (Beckman and Buchanan, 1995; Kim et al., 2017).

High knee extension moments during landing are associated with increased risk of ACL injuries (Alentorn-Geli et al., 2009). This injury mechanism is prevalent among female athletes (Karita et al., 2017). The primary reason is that women typically have wider hips, which leads to a larger Q-angle (the angle between the lateral femoral axis and the tibial axis) compared with men (Mizuno et al., 2001). An increased Q-angle enhances the lateral pulling force of the quadriceps, resulting in greater knee extension torque (Heiderscheit et al., 2000). Additionally, women’s weaker muscle strength and neuromuscular control may contribute to increased knee extension torque during landing (Hewett et al., 2005). Cadaver studies have shown that intense quadriceps contractions can cause ACL ruptures (DeMorat et al., 2004). Furthermore, during high-speed eccentric contractions and quadriceps activation during landing, the ACL is subjected to greater forces compared with those observed during maximum isometric contractions of the quadriceps (Griffin et al., 2000). Previous research also supported our findings, indicating that patients with CAI exhibited a higher quadriceps/hamstring co-activation ratio during inclined plane landings compared with healthy individuals (Li et al., 2017). During landing, the hamstring’s force is diminished, and the quadriceps play a dominant role. Previous studies have shown that greater hamstring activation can reduce the ACL load exerted by the quadriceps and provide dynamic knee stability by resisting anterior tibial translation, lateral tibial translation, and transverse tibial rotation (Renström et al., 1986; Withrow et al., 2008). Therefore, the increased knee extension moment observed during landing in patients with CAI may be a potential mechanism leading to ACL injuries in this population.

Previous research has revealed that the contraction of the knee and hip extensors does not decrease VGRF during the landing contact phase; by contrast, the associated energy is transferred to the bones and ligaments, thereby increasing joint contact stress and the risk of ACL and meniscal injuries (Mills et al., 2009). In addition, this phenomenon has been observed in the biomechanical factors related to ACL injury and knee joint energy absorption. At the terminal phase of landing, knee joint energy absorption is inversely related to VGRF and hip extension moments and directly related to peak hip flexion angles (Norcross et al., 2010). These findings indicated that high hip extension moments and reduced hip flexion angles could lead to insufficient knee joint energy dissipation capacity, producing high ACL loads and increasing ACL injury risks (Alentorn-Geli et al., 2009; Norcross et al., 2010).

Overall, the biomechanical pattern of sagittal plane landings in the CAI group, characterized by increased knee and hip extension torques and reduced hip flexion angles, may provide a potential mechanism for the heightened risk of individual ACL injuries following CAI.

### 4.2 Frontal plane kinematics of the lower limb

Contrary to our expectations, no significant differences in hip abduction angles and knee abduction angles were observed between patients with CAI and the healthy group. Our review included four studies on hip abduction angles (Brown et al., 2011; Jeon and Park, 2021; Sagawa et al., 2024; Terada et al., 2015) and three on knee abduction angles (De Ridder et al., 2015; Jeon and Park, 2021; Lee et al., 2017). The collected data generally showed similar knee and hip abduction angles between patients with CAI and healthy individuals during jump-landing tasks, with only one study reporting a reduced knee abduction angle (Jeon and Park, 2021) (Figure 3). Therefore, our meta-analysis found no significant differences between the two groups. During landing tasks, patients with CAI and healthy individuals experienced similar frontal plane kinematics.

Excessive knee abduction angles and knee abduction torques during jump landing are known critical factors influencing ACL injuries (Hewett et al., 2005; Olsen et al., 2004; Quatman and Hewett, 2009). A prospective cohort study of 205 female athletes found that those who suffered ACL injuries had previously exhibited larger knee abduction angles, smaller hip abduction angles, and greater knee abduction torques than their counterparts, These variables were considered predictive of ACL injuries (Hewett et al., 2005). Therefore, avoiding large knee abduction angles and torques and small hip abduction angles during landing may help reduce ACL injury risks in patients with CAI. Given the limited research on related frontal plane dynamics, our study did not include metrics of knee abduction/adduction torques linked to ACL injuries. Future research should incorporate outcomes related to frontal plane biomechanics to elucidate the relationship between the landing frontal plane biomechanics of patents with CAI and ACL injury risks.

### 4.3 Impact loading

Our review included seven studies on peak VGRF (Caulfield and Garrett, 2004; De Ridder et al., 2015; Jeon and Park, 2021; Lee et al., 2017; Watabe et al., 2022; Watanabe et al., 2021; Zhang et al., 2012) and three on vertical loading rates (De Ridder et al., 2015; Jeon and Park, 2021; Lee et al., 2017) as measures of VGRF and loading rates. The results indicated that patients with CAI exhibited a comparable loading rate with healthy individuals, with an increased peak VGRF (Figure 4).

Previous reviews were consistent with our findings that is, patients with CAI have a high peak VGRF during landing (Jeon et al., 2021; Simpson et al., 2018), which has been identified as a non-contact ACL injury risk (Lin et al., 2012). A prospective study found that female athletes with knee injuries who later experienced ACL ruptures had a 20% higher peak VGRF than those without knee injuries and ACL damage (Hewett et al., 2005; Paterno et al., 2007). The maximum ACL load during landing tasks occurs at the moment of impact peak VGRF (Paterno et al., 2007; Watabe et al., 2022), with an increase in peak VGRF in patients with CAI leading to high ACL loading and great ACL injury risks. Therefore, minimizing the peak VGRF during landing may reduce ACL injury risks in patients with CAI.

The loading rate, as a factor contributing to ACL injuries during landing, has been noted in previous studies to be elevated in patients with CAI (Paterno et al., 2007; Simpson et al., 2018), Although this finding contradicted our results, no significant differences in loading rates were observed. This discrepancy may stem from our dataset, which included only a few studies on loading rates, highlighting the need for further research on this phenomenon.

Other impact loading–related metrics could be used to distinguish between abnormal lower limb impact loads in patients with CAI and healthy individuals. Frontal plane ground reaction forces (Hewett et al., 2009), sagittal plane ground reaction forces (Lin et al., 2012), and symmetry of landing forces are all associated with increased risks of ACL injuries (Paterno et al., 2007; Paterno et al., 2010; Paterno et al., 2011). However, these metrics were not included in this study. In the future, we will to incorporate more indicators of impact loading to clarify the relationship between impact loading during landing in patients with CAI and ACL injury risks.

### 4.4 Trunk mechanics

Low trunk flexion is associated with ACL injuries (Griffin et al., 2000). A cross-sectional cohort study found that female subjects with ACL injuries exhibited less trunk flexion compared with a control group of women, aligning their trunk with their legs in a way that increases valgus and axial forces (Hewett et al., 2009). High trunk flexion angles can prolong the time to reach peak VGRF, reduce landing impulse, and effectively absorb VGRF, decreasing the load on the ACL (Paterno et al., 2007; Watabe et al., 2022). However, this phenomenon not observed in patients with CAI. This review included two studies on this topic (Brown et al., 2011; Watabe et al., 2022). Watabe et al. (2022) studied the differences in lower limb and trunk biomechanics during active and passive single-leg landings between patients with CAI and healthy individuals, demonstrating that patients with CAI exhibit high trunk flexion angles in active and passive settings. Brown et al. (2011) examined the variability in movements during single-leg landing tasks among FAI, MAI, and healthy groups, and they found no significant differences in trunk flexion angles between patients with FAI/MAI and healthy individuals. Our review included these two studies (Brown et al., 2011; Watabe et al., 2022), and publication bias was statistically tested using Egger’s test (*p* = 0.007), indicating that the likelihood of publishing significant results may be higher than that for nonsignificant results. The presence of this bias necessitates a cautious approach in understanding and interpreting the relationship between trunk flexion and ACL injuries. Future research should utilize a broader range of data sources to ensure the comprehensiveness and balance of the findings. Hence, the clinical significance of the current findings remains uncertain.

**FIGURE 5:**
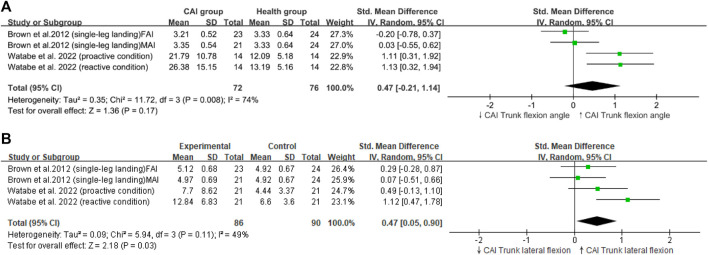
Trunk mechanics; **(A)**: Trunk flexion, **(B)**: Trunk lateral flexion.

Furthermore, our review revealed that patients with CAI exhibited greater trunk lateral flexion than healthy controls (Figure 5). A prospective study of over 900 athletes supported this finding, showing that female athletes with ankle injuries (such as CAI and lateral ankle sprains) demonstrate greater trunk sway compared with uninjured athletes (Beynnon et al., 2006). Insufficient neuromuscular control of the body’s core and decreased dynamic postural stability may contribute to increased trunk lateral flexion angles during landing (Kibler et al., 2006). The limitations associated with CAI have been shown to impair dynamic postural stability and neuromuscular control during unilateral jump landings, leading to sensorimotor function impairment (Caulfield et al., 2004; Caulfield and Garrett, 2002; Gribble and Robinson, 2009). Impaired neuromuscular control, especially in the hip muscles, is often observed in patients with CAI, who typically exhibit symptoms of deficient neuromuscular control (Bullock-Saxton et al., 1994; McCann et al., 2017; Terada et al., 2016). Therefore, the increased trunk lateral flexion observed in patients with CAI was due to decreased dynamic postural stability and inadequate neuromuscular control of the hip. Zazulak et al. (2007) suggested that trunk lateral displacement is a strong predictor of knee ligament injury, including ACL injuries. Their study of athletes over 3 years found that those with knee, ligament, or ACL injuries exhibit greater trunk displacement in all planes compared with uninjured athletes, identifying lateral displacement as the strongest predictor of ligament injuries. Trunk lateral flexion causes the lateral ground reaction force vector to shift sideways and, having a longer lever arm relative to the knee joint center, directly increases the likelihood of knee abduction motion and torque, thereby elevating the risk of ACL injuries (Hewett et al., 2009). Therefore, the landing mechanics of patients with CAI characterized by pronounced trunk lateral flexion could be factor contributing to the increased risk of ACL injuries in this population.

### 4.5 Limitation

Our study has the following limitations and weaknesses. First, the current research includes young athletes (under 27 years old) from various sports and regularly exercising individuals. Therefore, our results may not be applicable to other populations with different ages and levels of physical activity. Second, given limited previous research on horizontal biomechanical variables related to ACL injuries and knee coronal plane torque as outcome measures, we were unable to perform meta-analyses on these aspects. Third, many risky movements,such as cutting and sudden stopping, can lead to ACL injuries; considering the heterogeneity and representativeness of injury movements, we selected only landing actions for analysis. Fourth, our results only indicate the differences in landing biomechanics between individuals with CAI and healthy controls during landing tasks. We cannot ascertain whether these differences would vary with different experimental protocols. Even for the same type of jump-landing tasks, the implementation protocols were not uniform. We also observed variations in platform height and single versus double foot landings, which led to high heterogeneity in some outcome indicator analyses. Lastly, each meta-analysis included only a small number of studies; Thus, the results of this systematic review should be interpreted with caution.

## 5 Conclusion

This study confirmed the association between CAI and increased sagittal plane kinetics, reduced hip flexion angles, increased peak VGRF, and increased trunk lateral flexion, all of which are related to a heightened risk of ACL injuries during landing tasks. These findings provide a basis for improving the understanding of ACL injury risks during landing tasks in individuals with CAI. This knowledge can guide future preventative measures and rehabilitation strategies to mitigate ACL injury risks in this population.

## Data Availability

The original contributions presented in the study are included in the article/Supplementary Material further inquiries can be directed to the corresponding author.
