# Supplementary material for: Does chronic ankle instability patients lead to changes in biomechanical parameters associated with anterior cruciate ligament injury during landing? A systematic review and meta-analysis
Source: Front Physiol. 2024 Aug 29;15:1428879. doi: 10.3389/fphys.2024.1428879 (PMC11391935; doi:10.3389/fphys.2024.1428879)

Supplementary Material

1 Appendix 1: Egger's regression tests

	95% CI	p
Peak VGRF	-6.072 to 1.102	0.149
Loading rate	-124.489 to 113.434	0.66
Hip flexion angle	-7.781 to 3.512	0.429
Knee flexion angle	-2.664 to 16.132	0.143
Ankle dorsiflexion angle	-4.257 to 8.848	0.463
Trunk flexion	7.014 to 14.765	**0.007***
Trunk lateral flexion	-4.761 to 51.557	0.07
Knee abduction angle	-31.283 to 6.527	0.129
Hip abduction angle	-27.274 to 2.612	0.143
Hip extension moment	-10.013 to 17.819	0.351
Knee extension moment	-1.609,13.968	0.076

2 Appendix 2: Funnel diagram of each index in this study.

A. Hip flexion angle

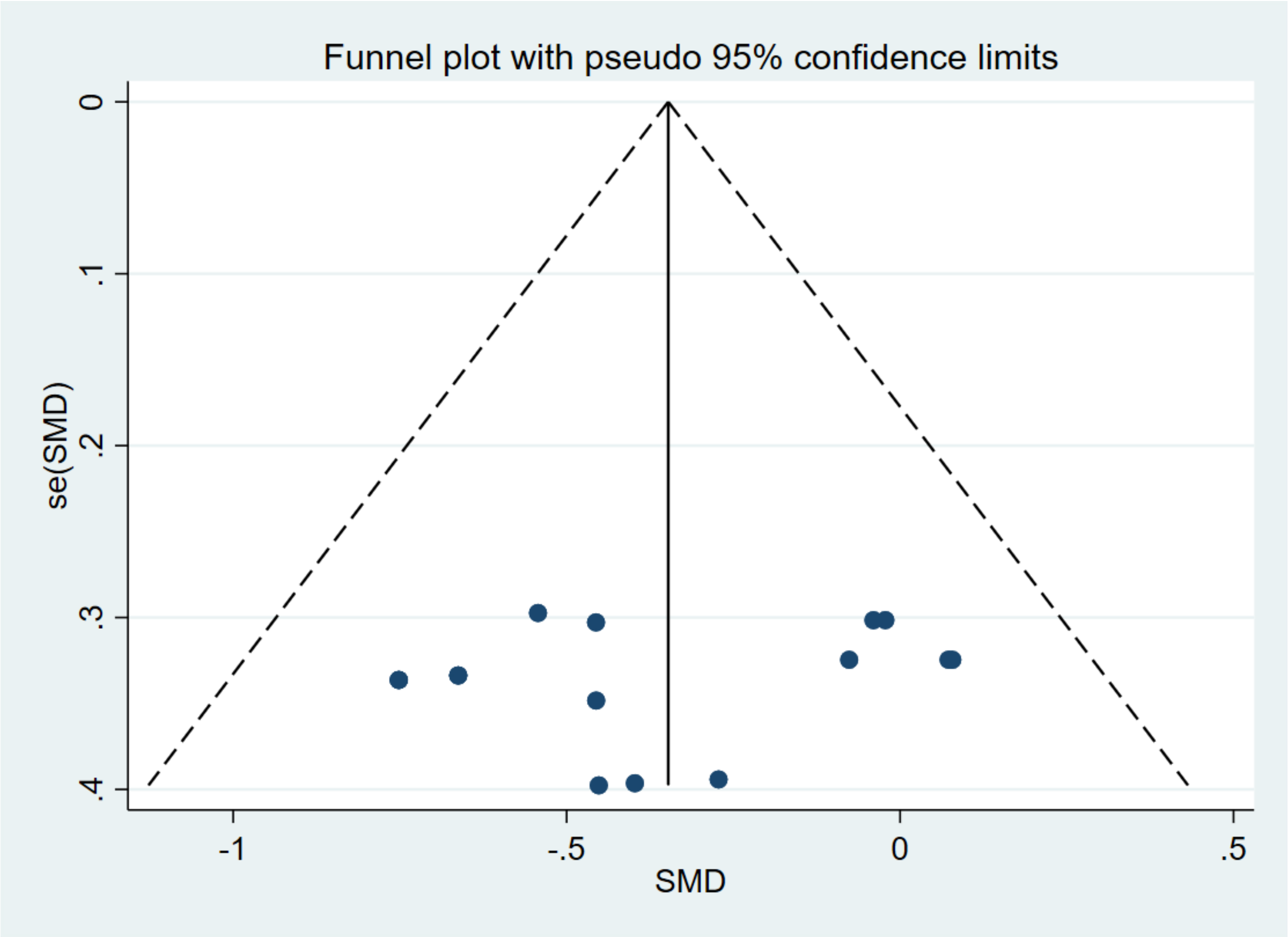

B. Knee flexion angle

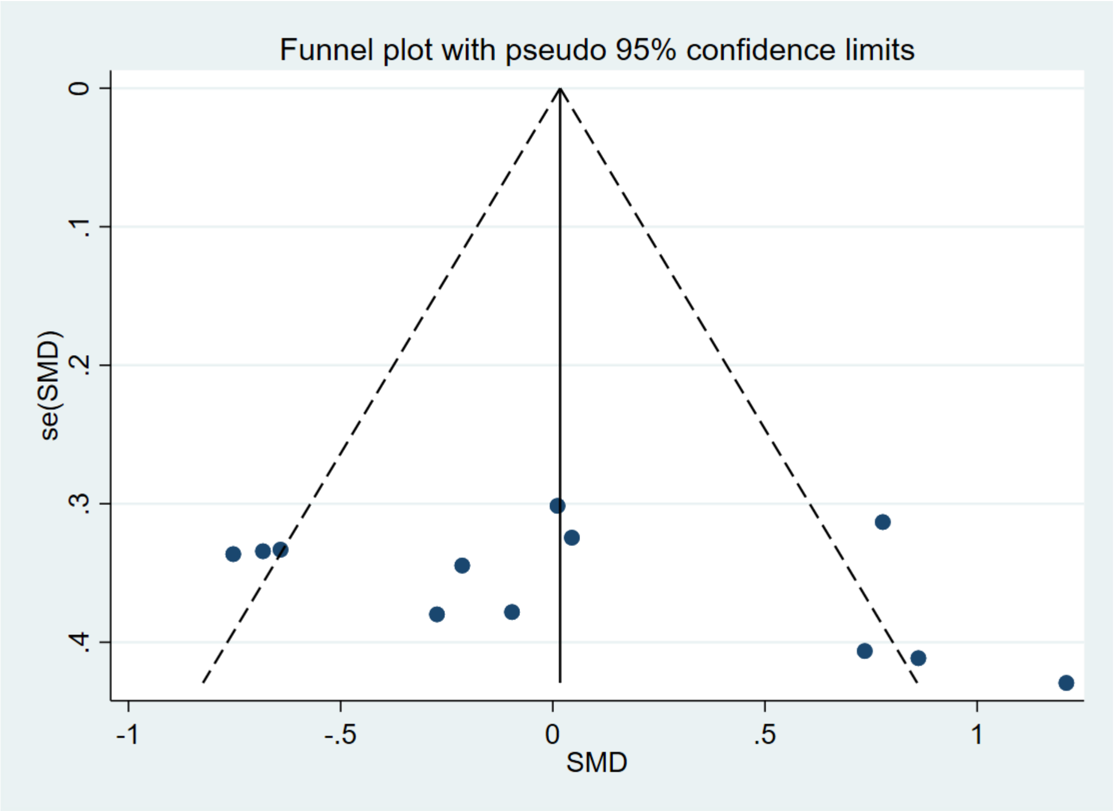

C. Ankle dorsiflexion angle

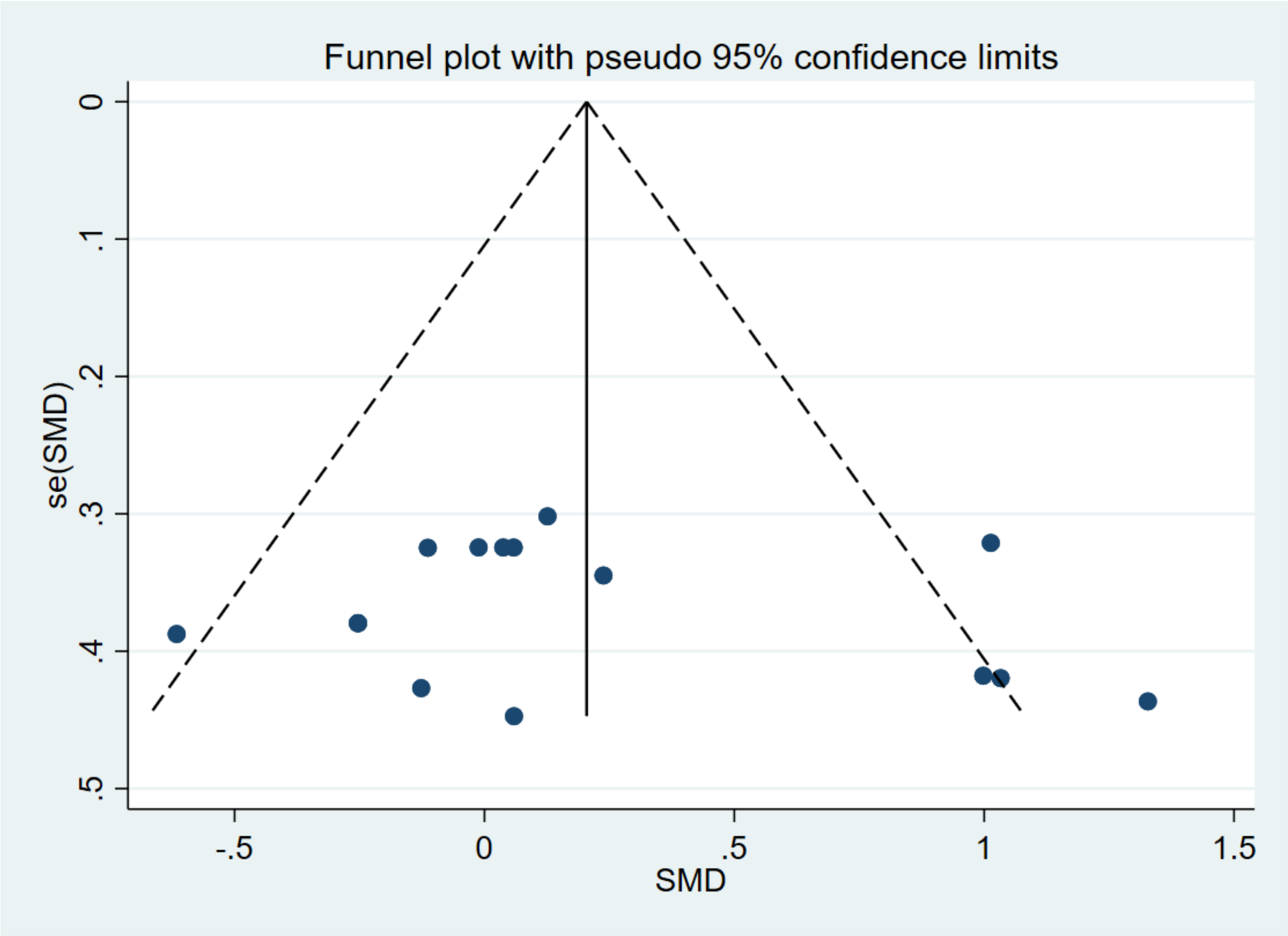

D. Hip extension moment

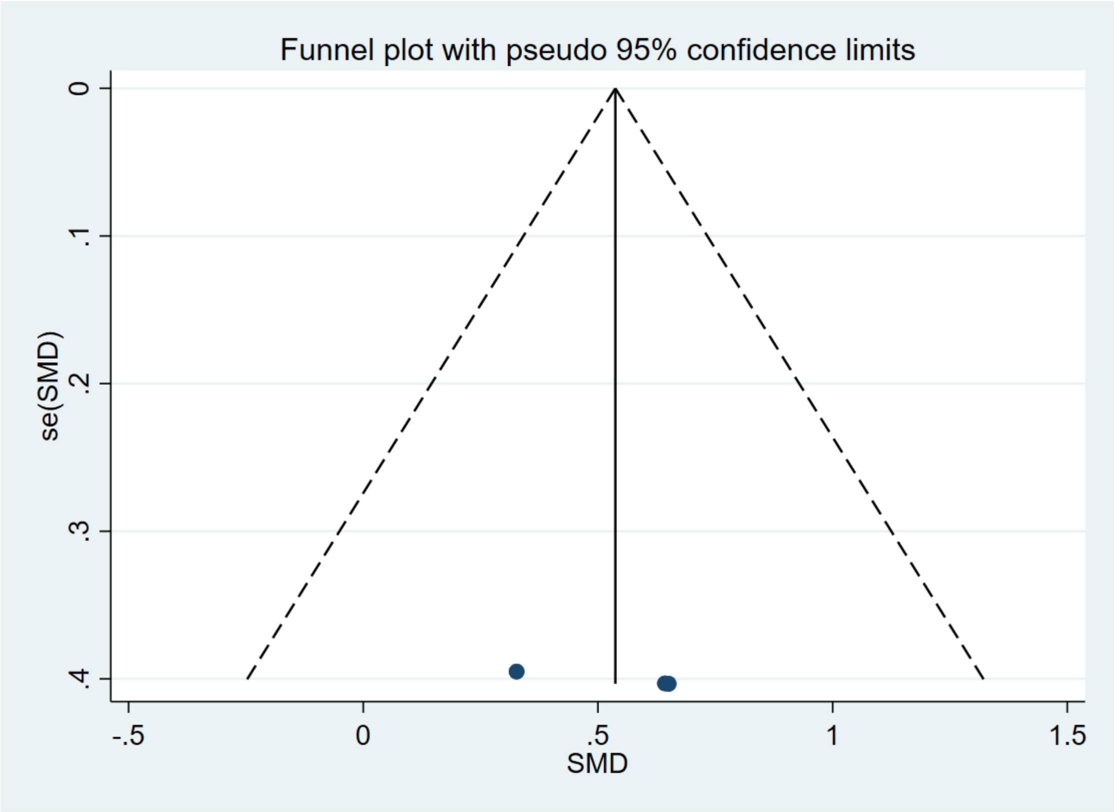

E. Knee extension moment

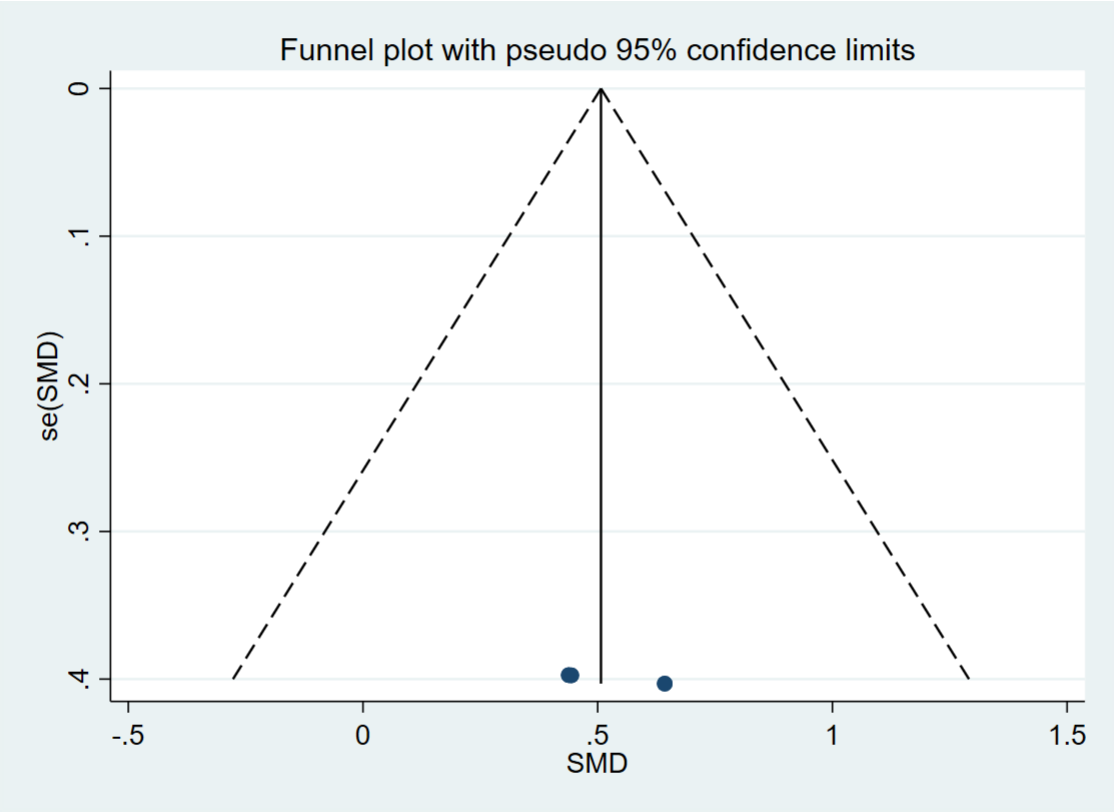

F. Hip abduction angle

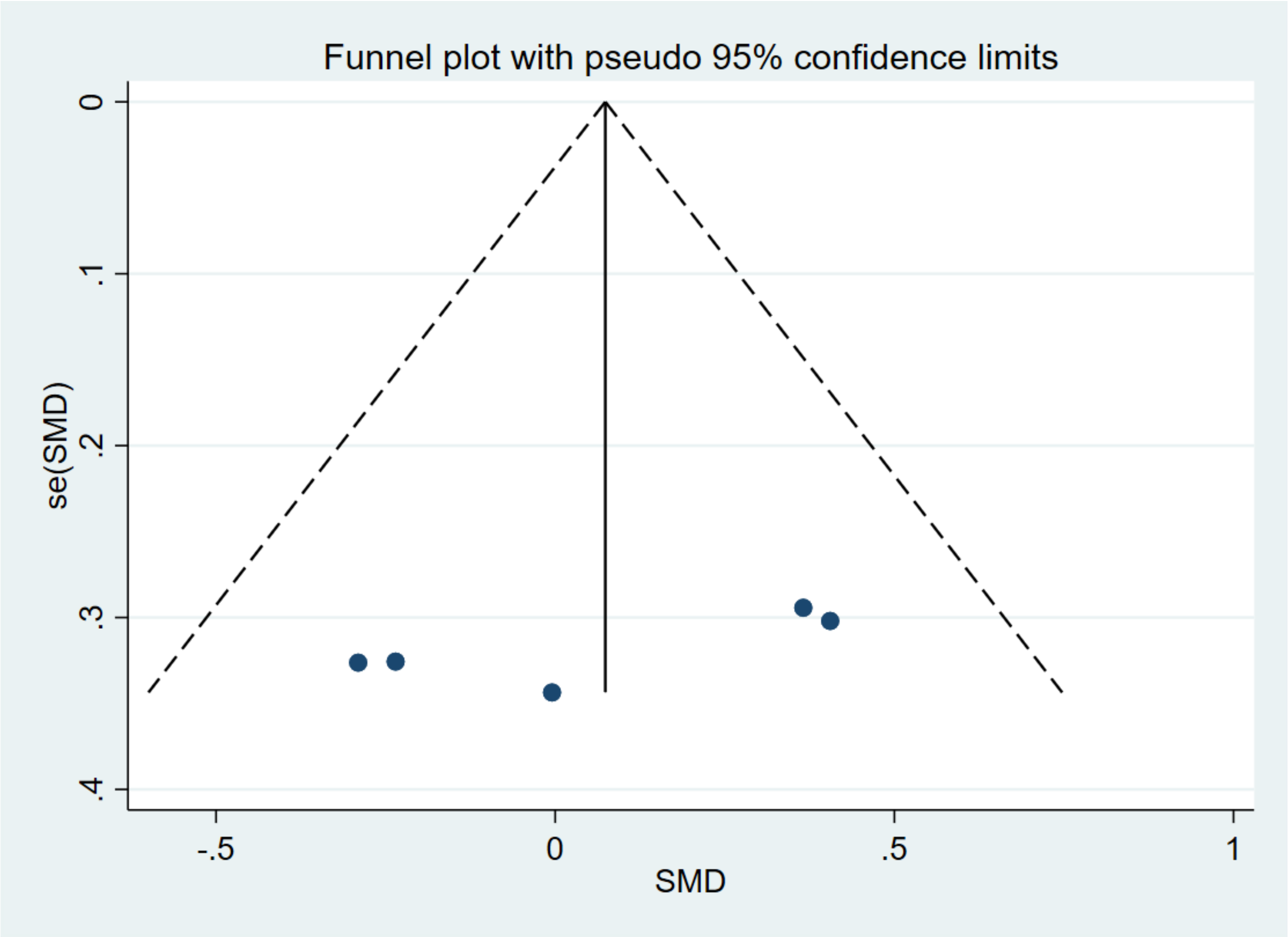

G. Knee abduction angle

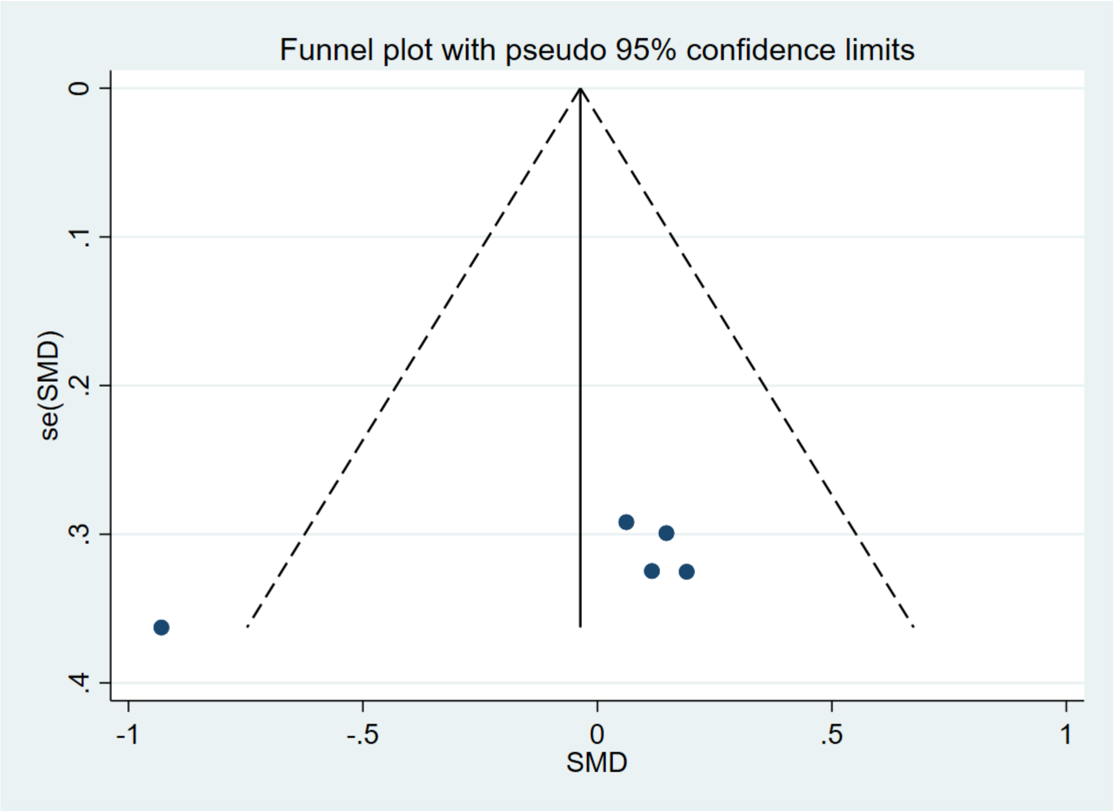
Funnel plot with pseudo 95% confidence limits
0
.1
.2
.3
.4
se(SMD)
-1
-.5
0
.5
1
SMD

H. Peak VGRF

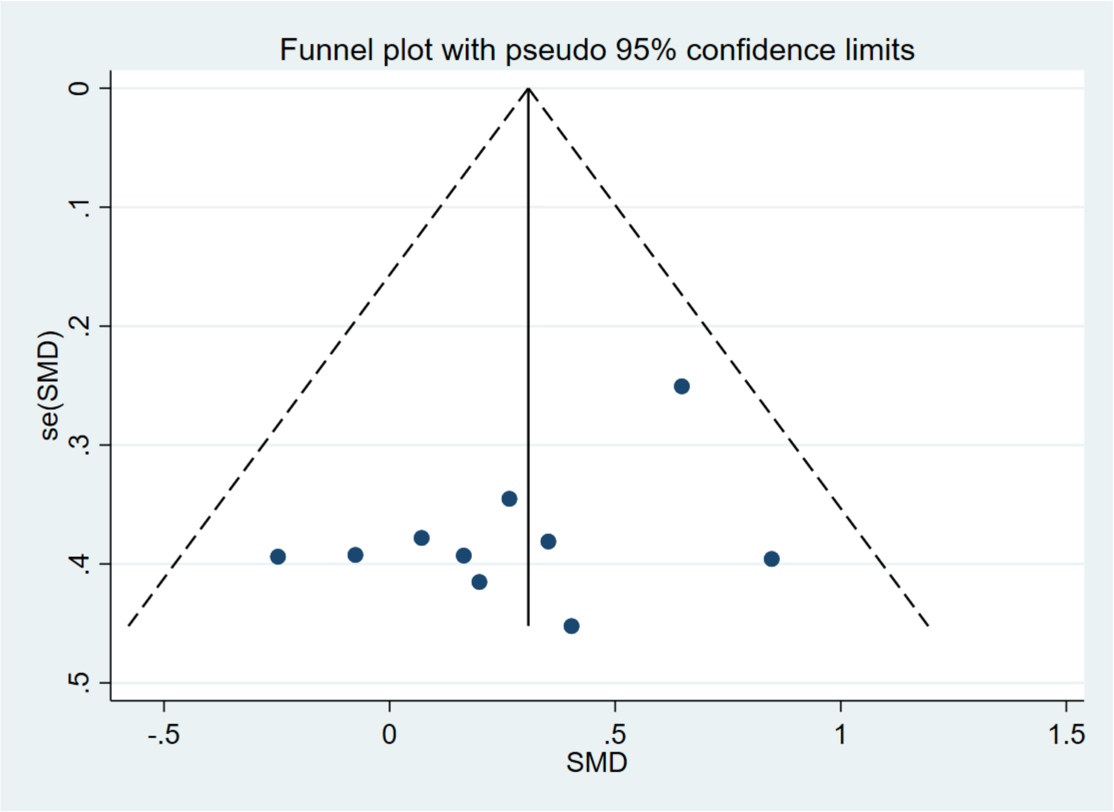

I. Loading rate

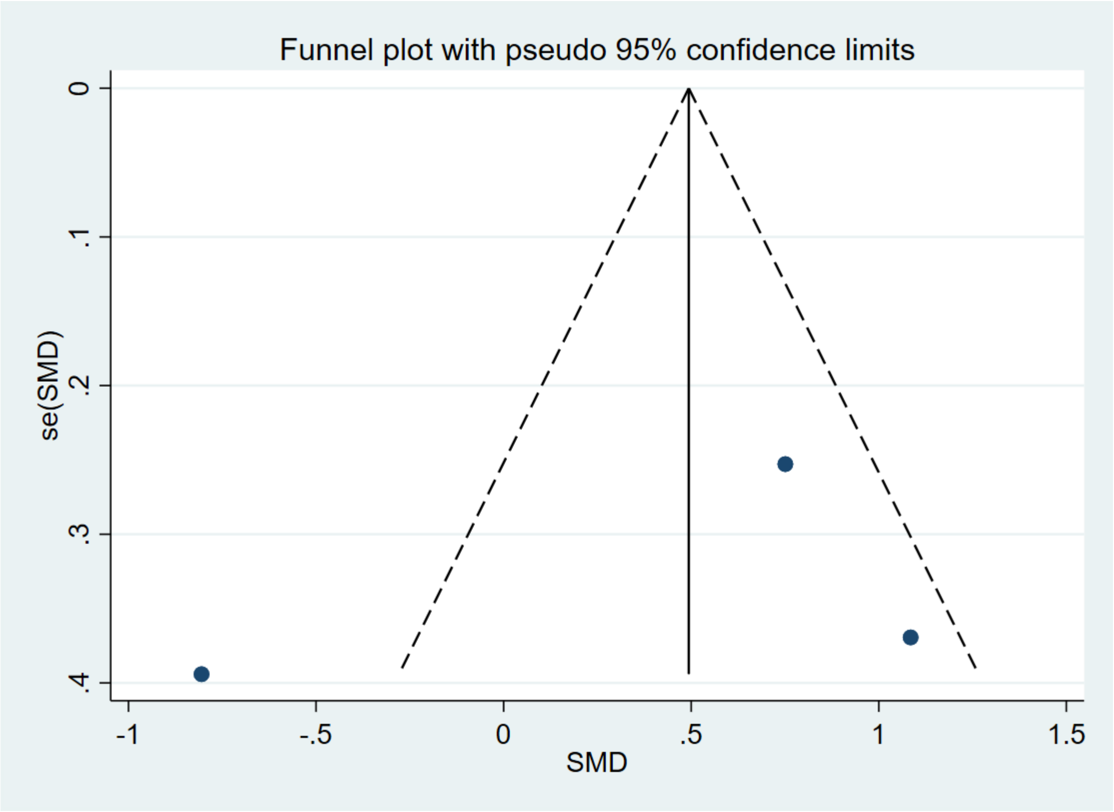

J.Trunk flexion

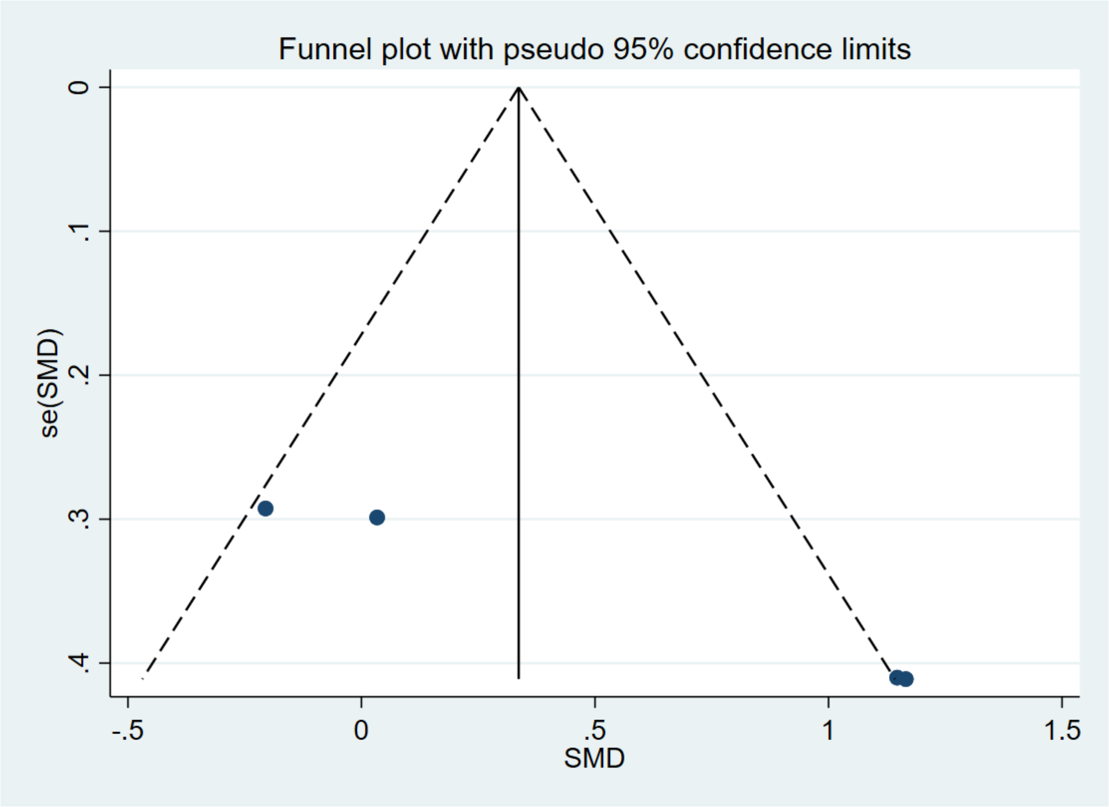

K. Trunk lateral flexion

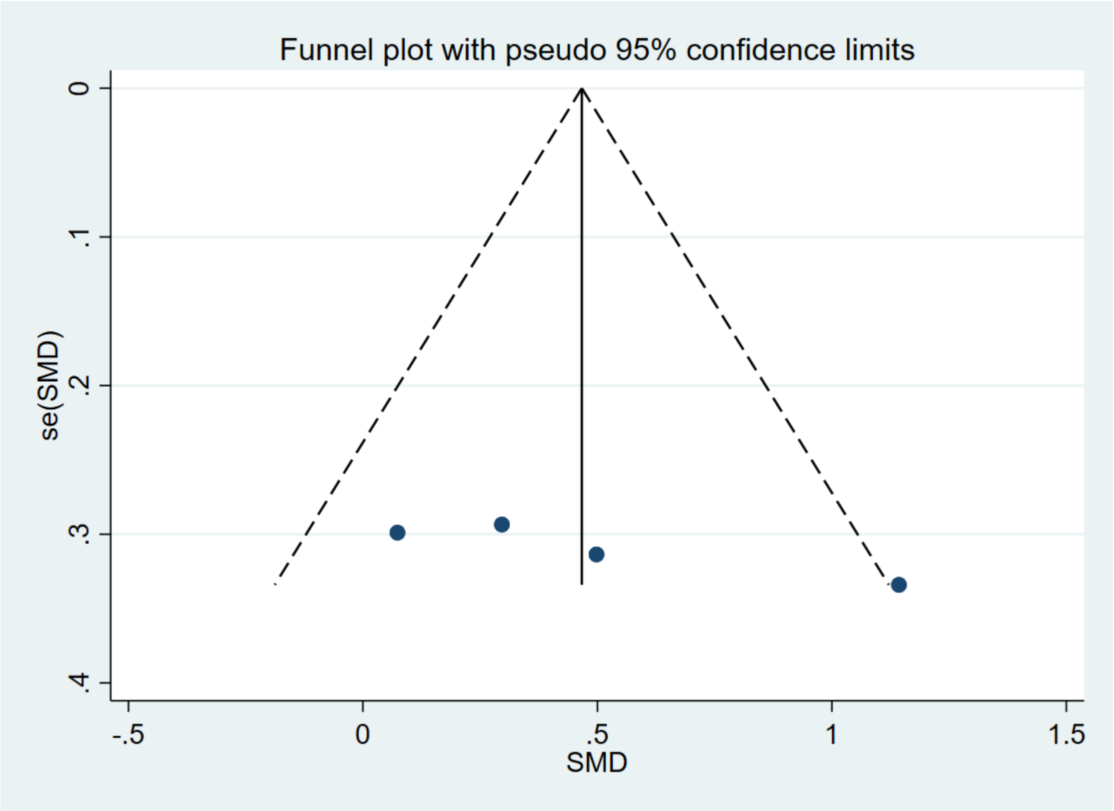

Appendix 4: Sensitivity test

A. Hip flexion angle

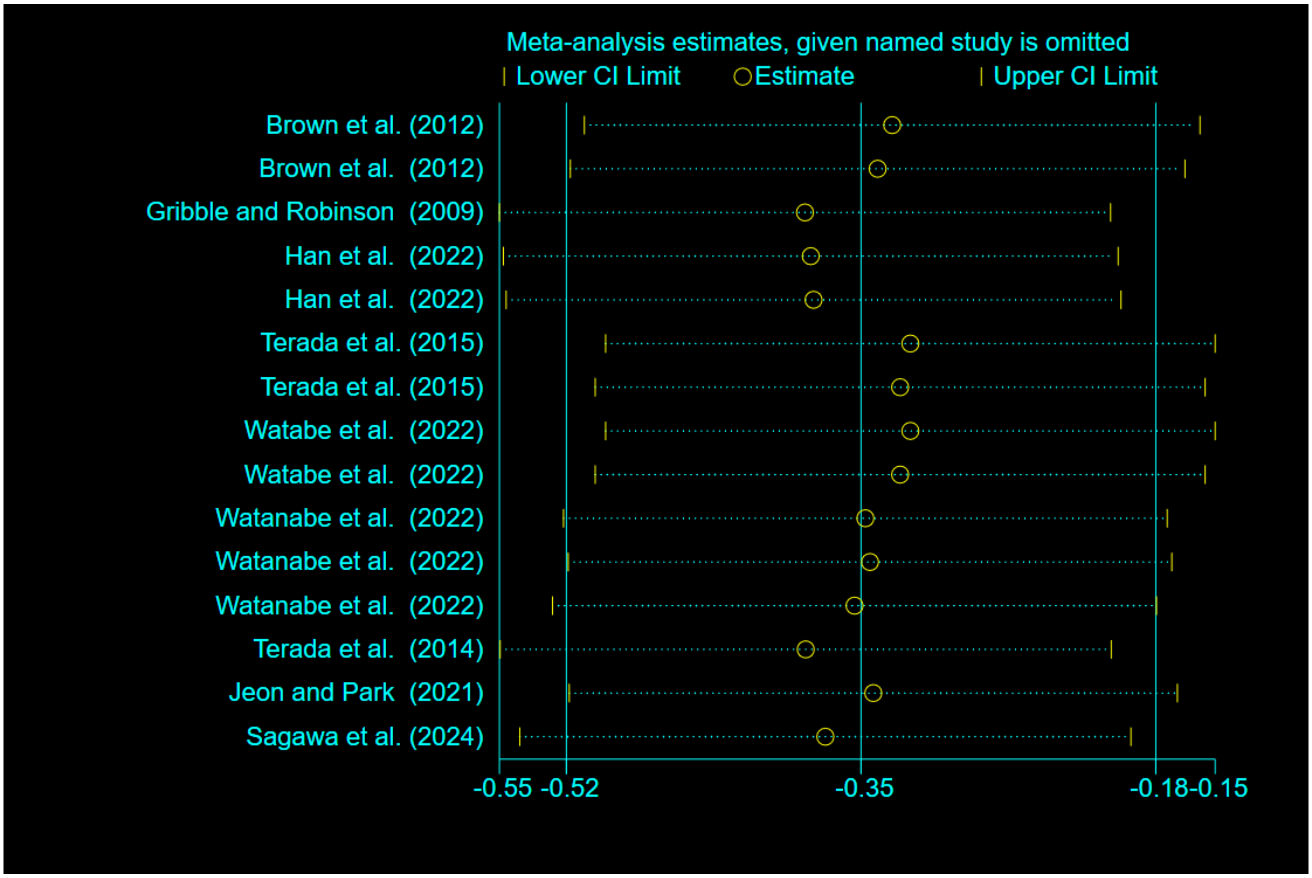

B. Knee flexion angle

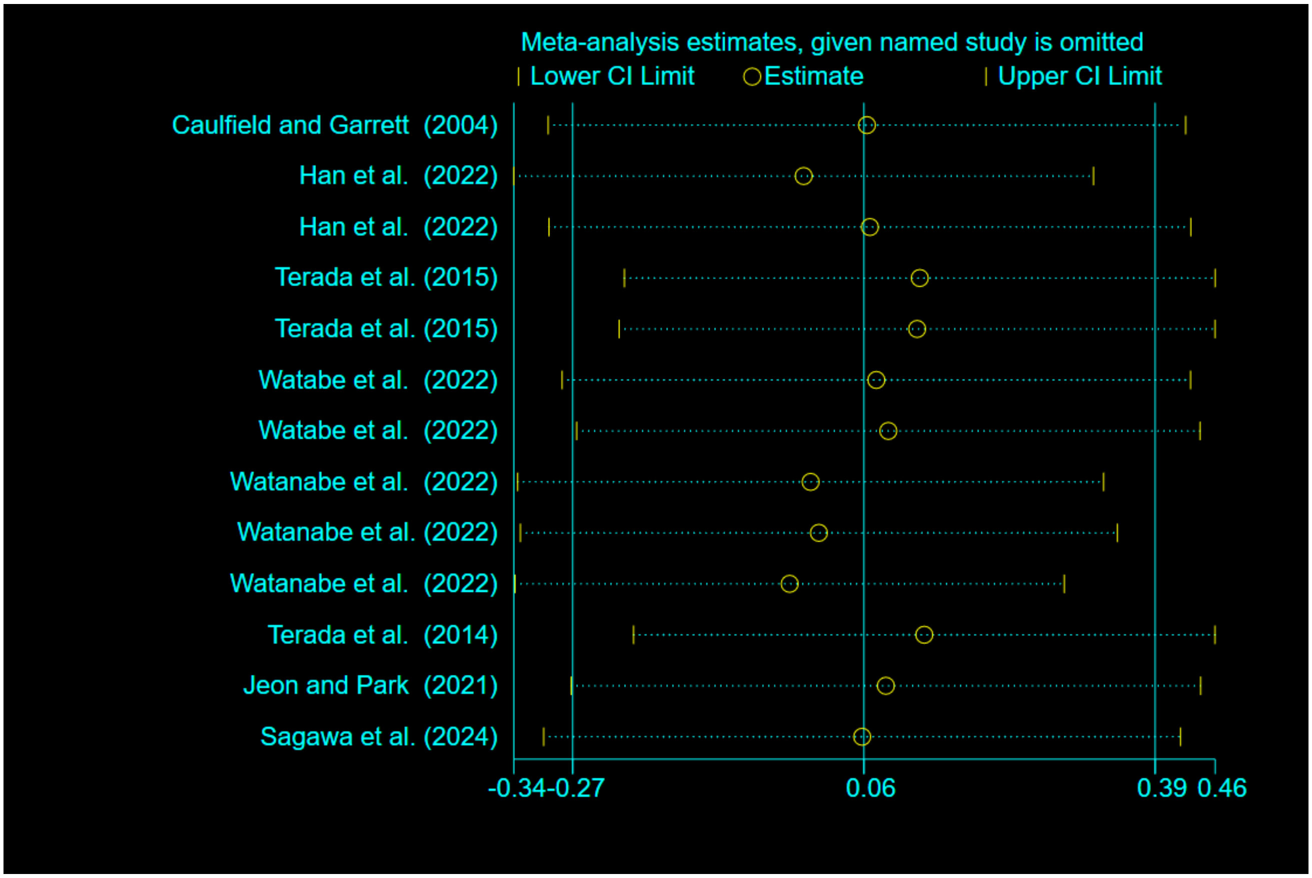

C. Ankle dorsiflexion angle

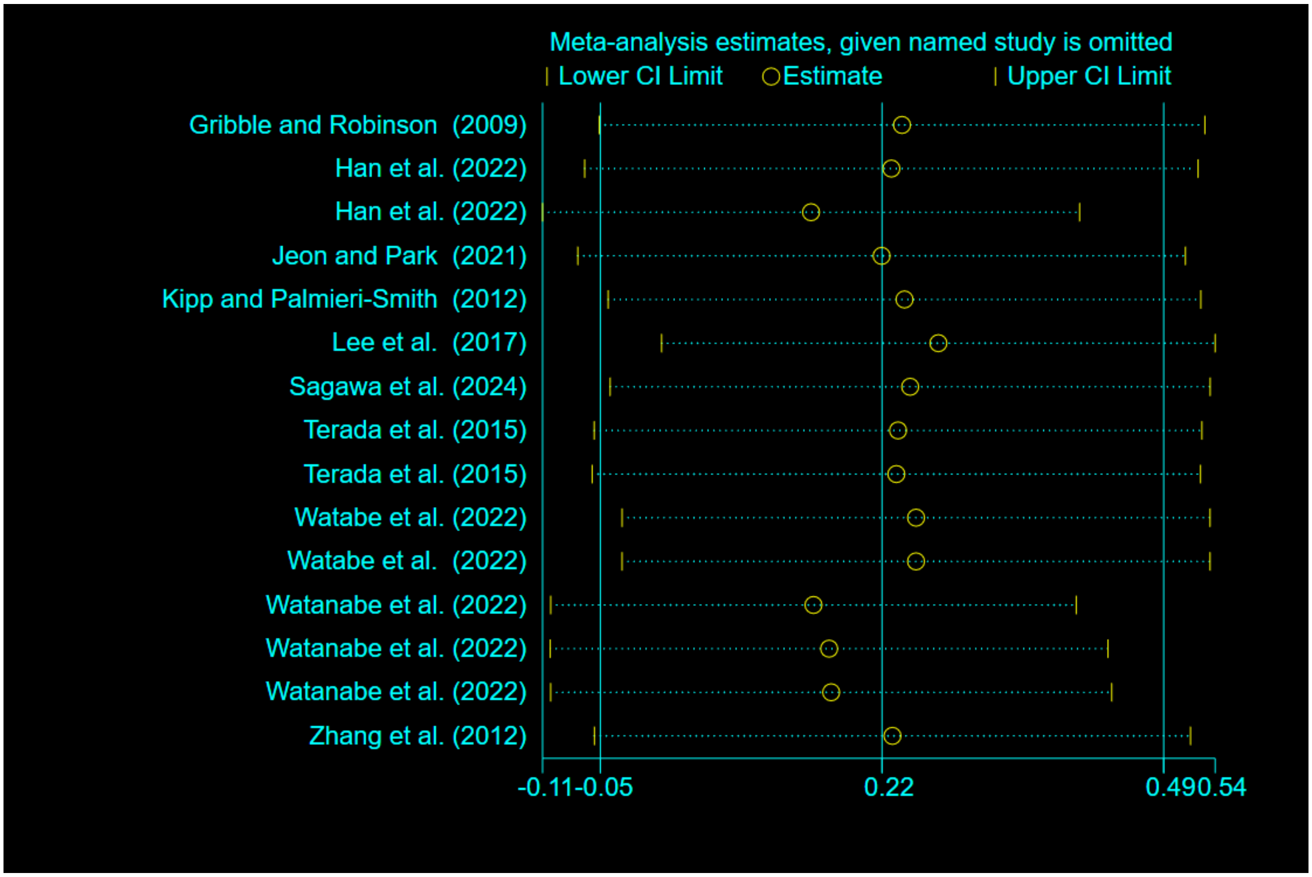

D. Hip extension moment

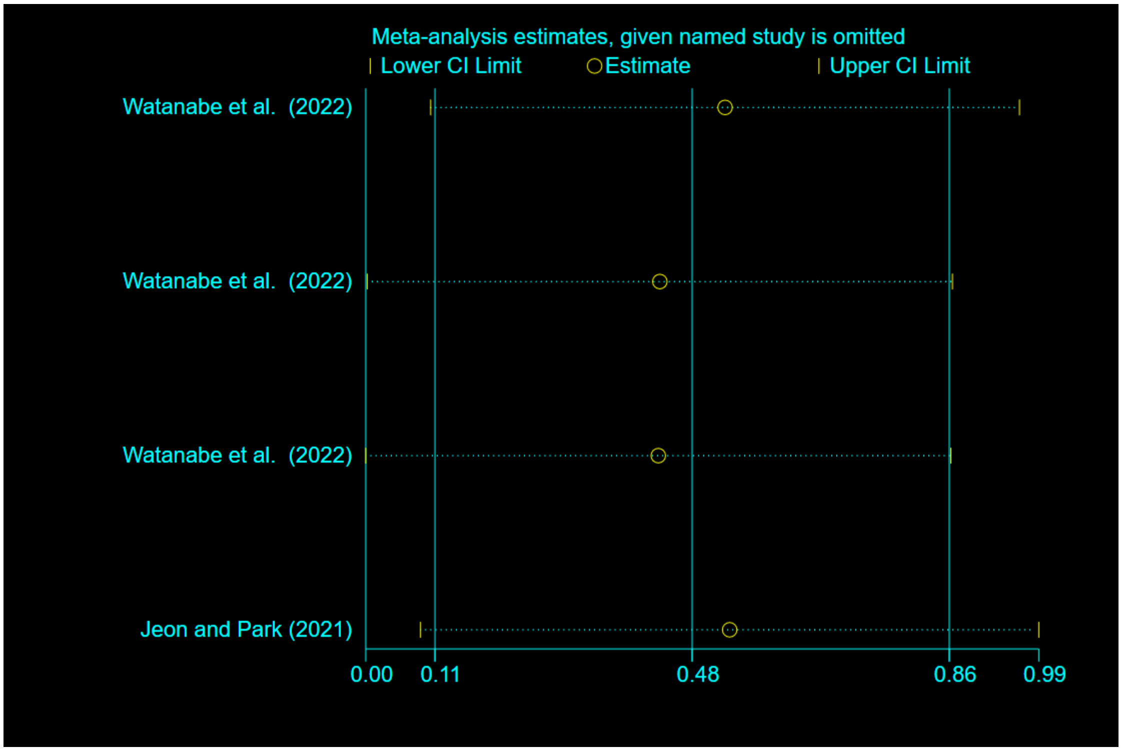

E. Knee extension moment

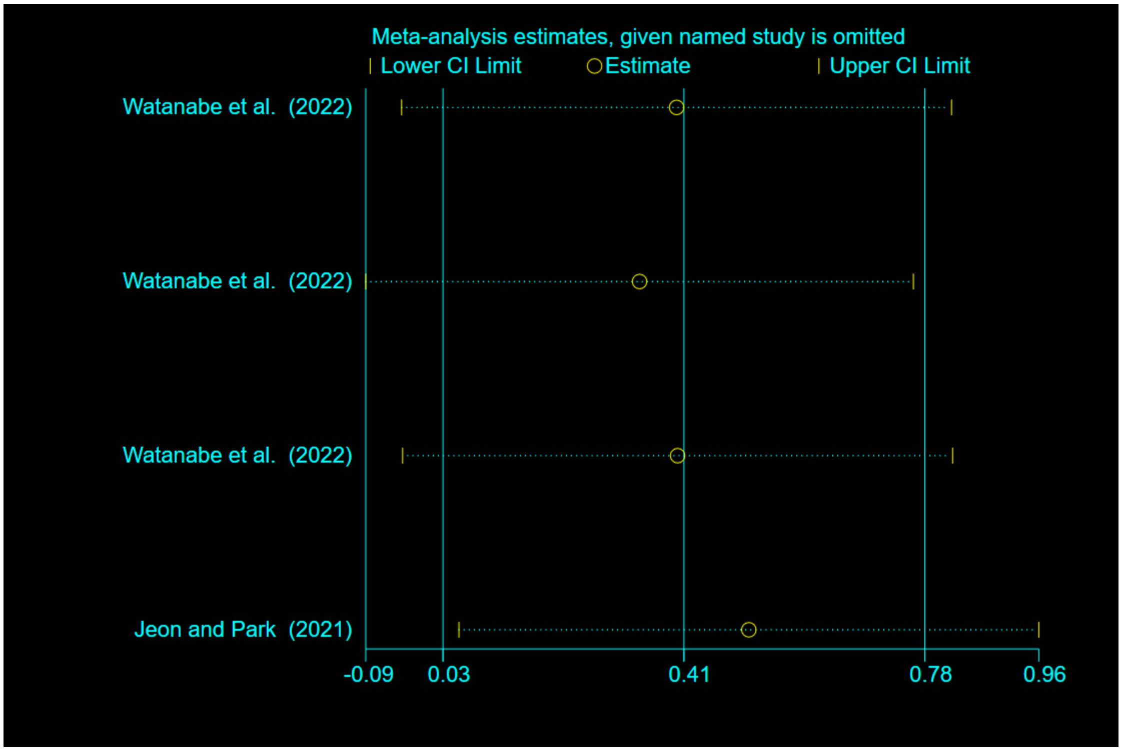

F. Hip abduction angle

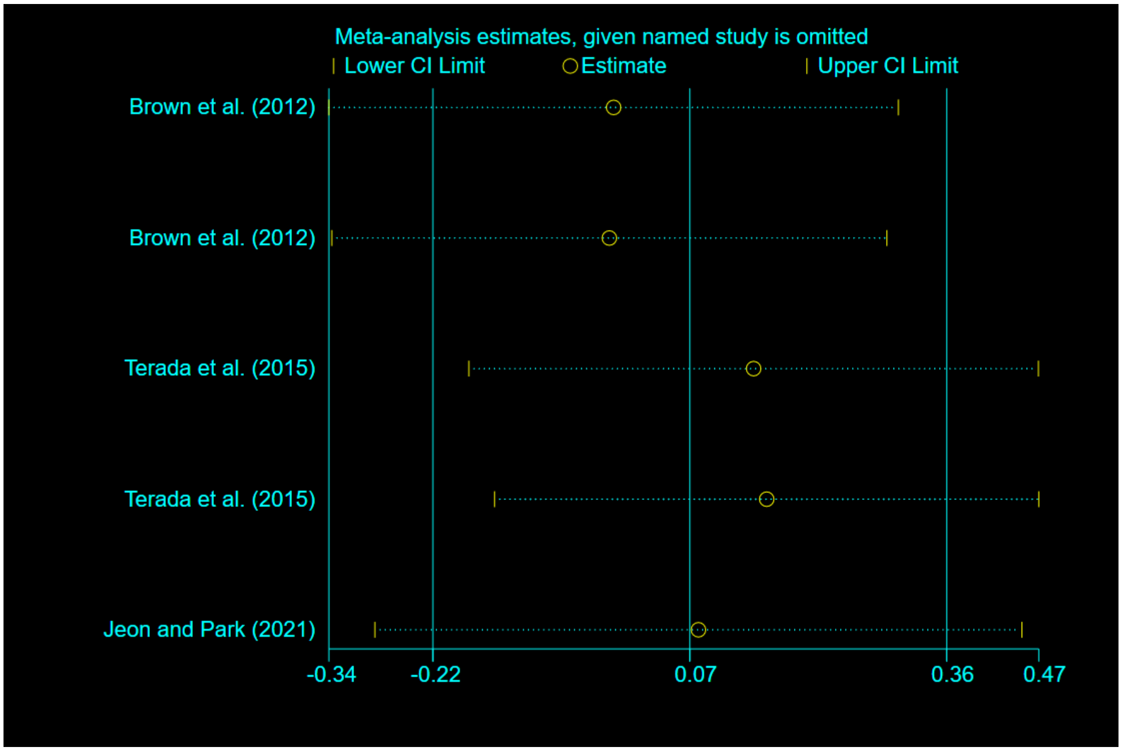

G. Knee abduction angle

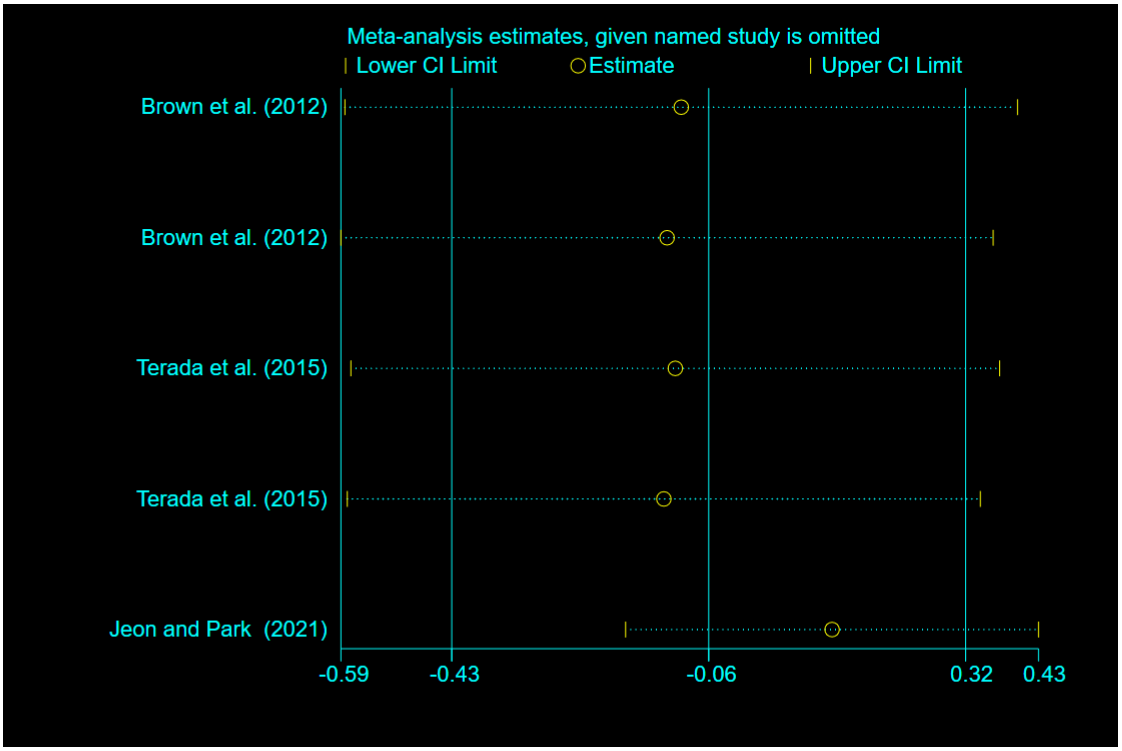

H.VGRF

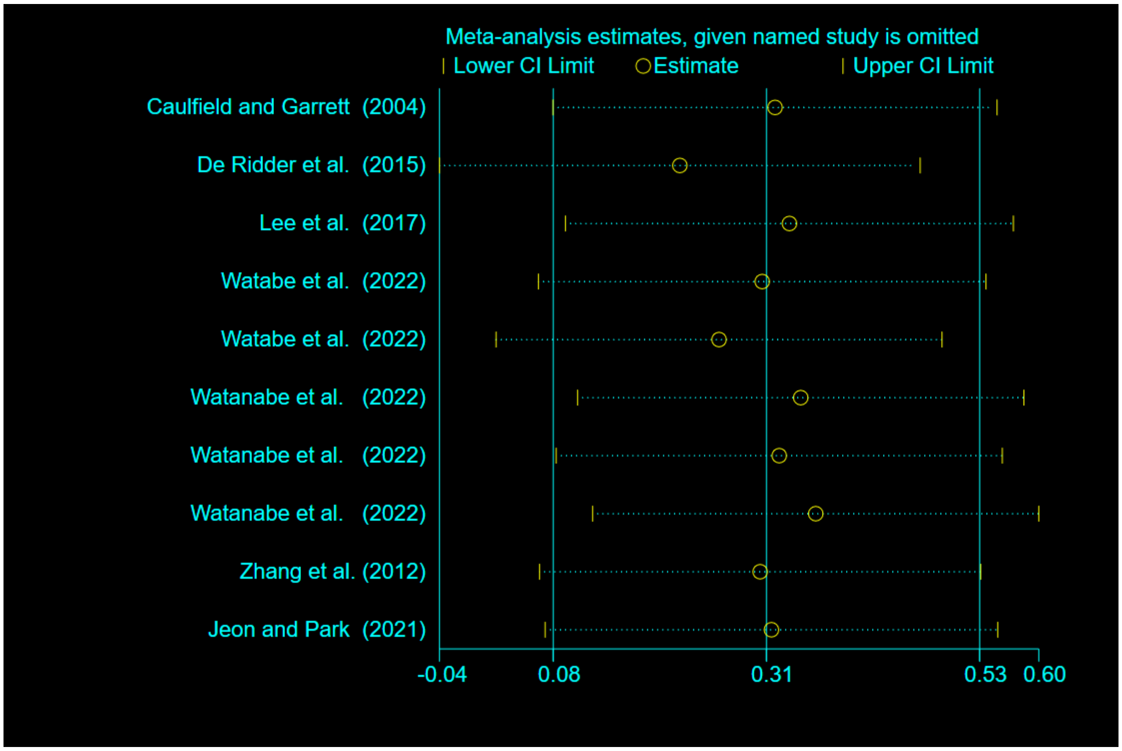

I. Loading rate

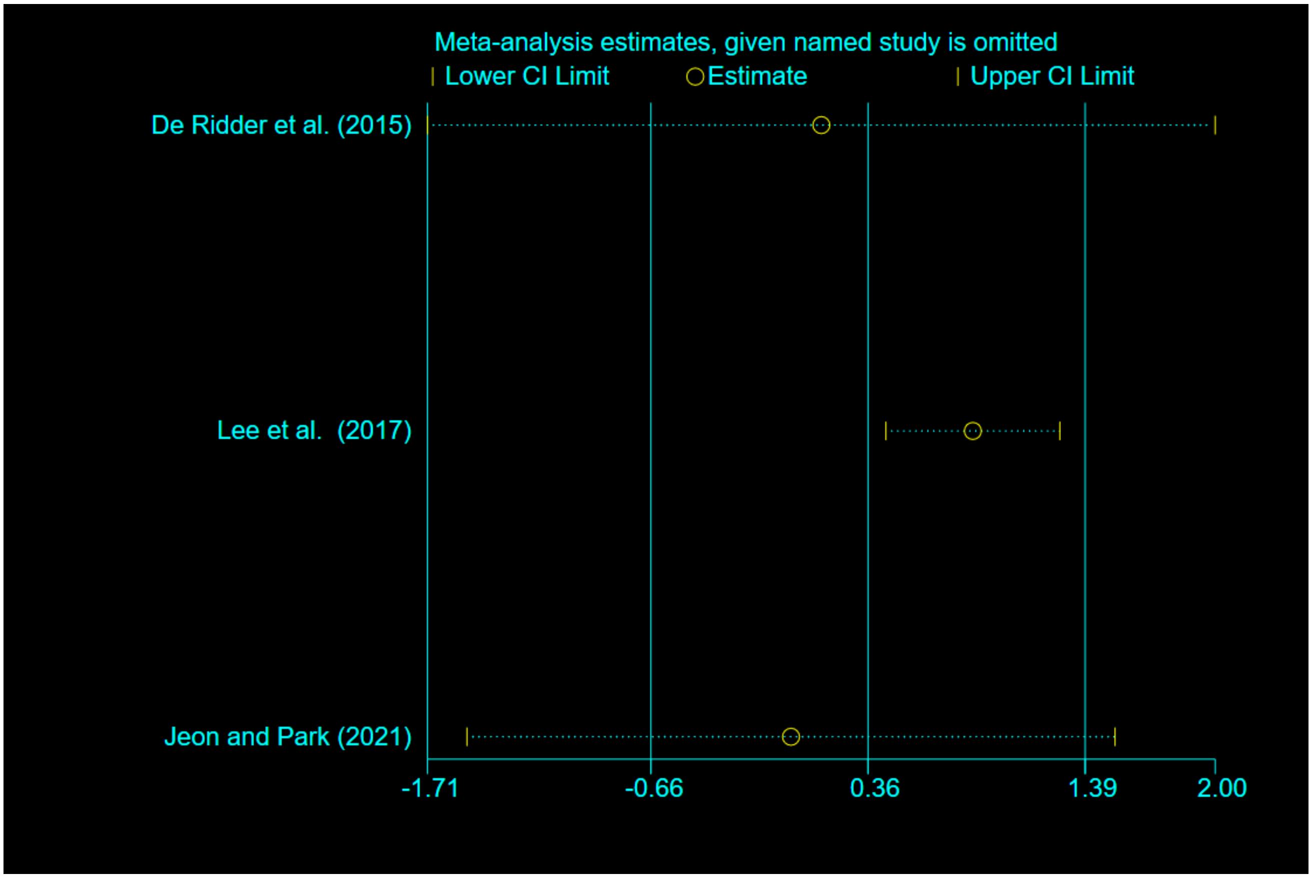

J.Trunk flexion

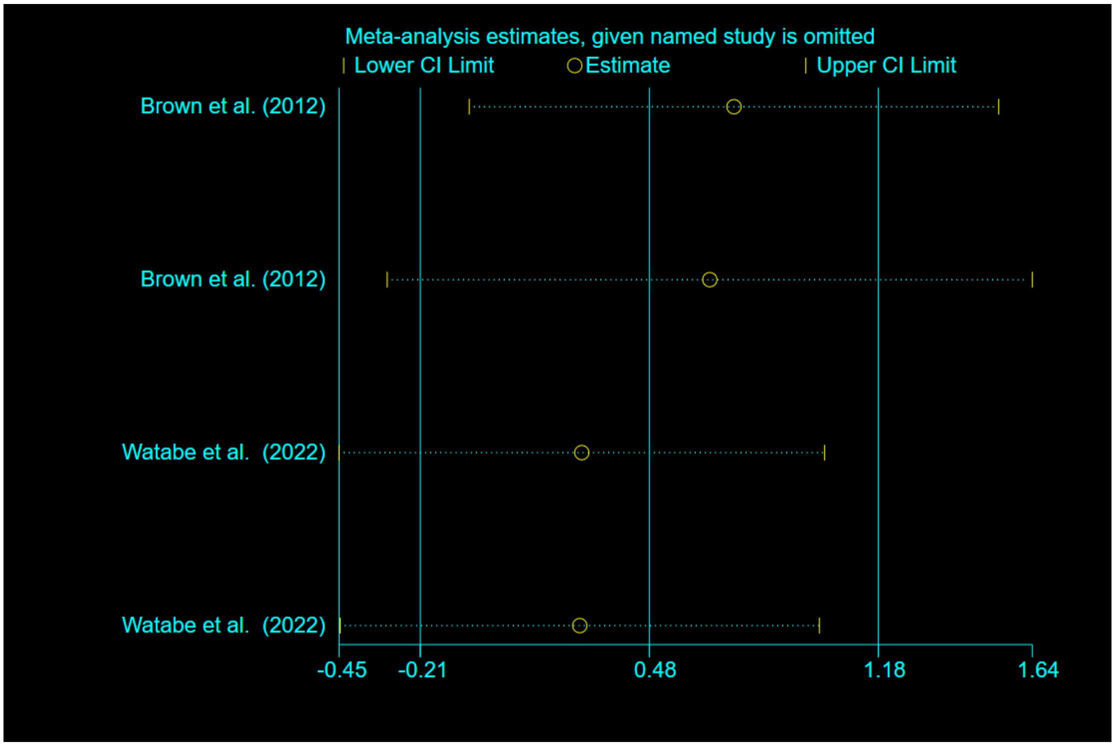

K.Trunk lateral flexion

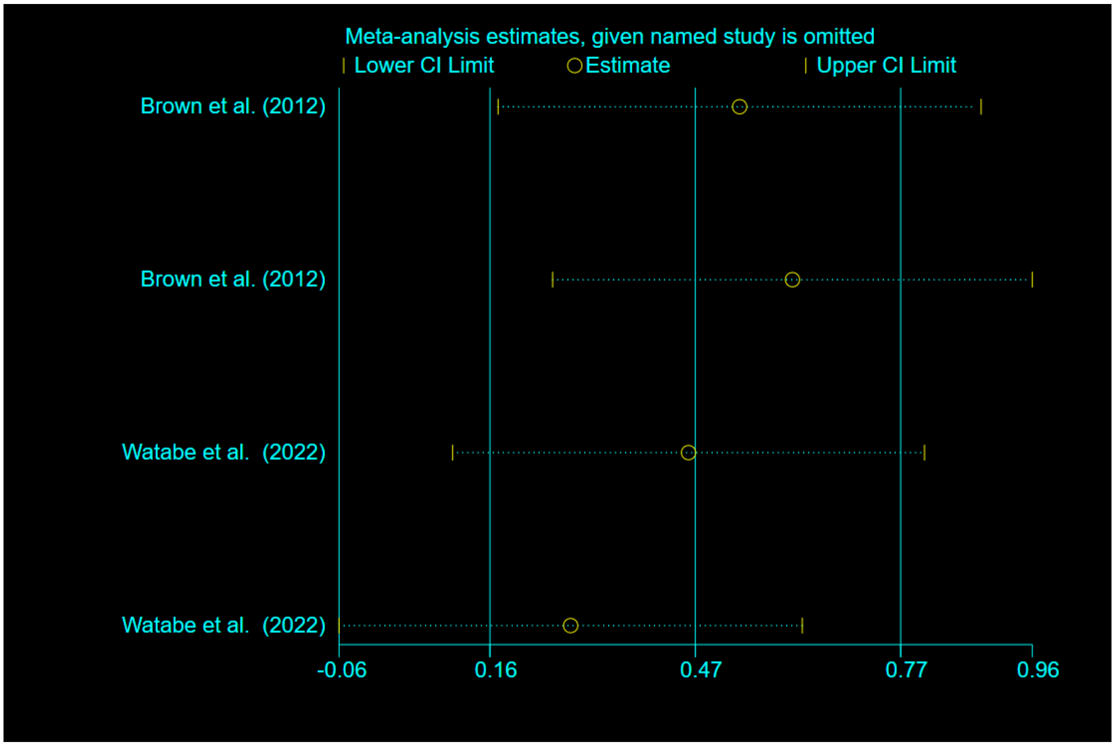

Supplement: Supplementary file 1 [file DataSheet1.PDF]
